# Supplementary figures and images for: Osteocalcin of maternal and embryonic origins synergize to establish homeostasis in offspring
Source: EMBO Rep. 2024 Jan 16;25(2):12. doi: 10.1038/s44319-023-00031-3 (PMC10897216; doi:10.1038/s44319-023-00031-3)

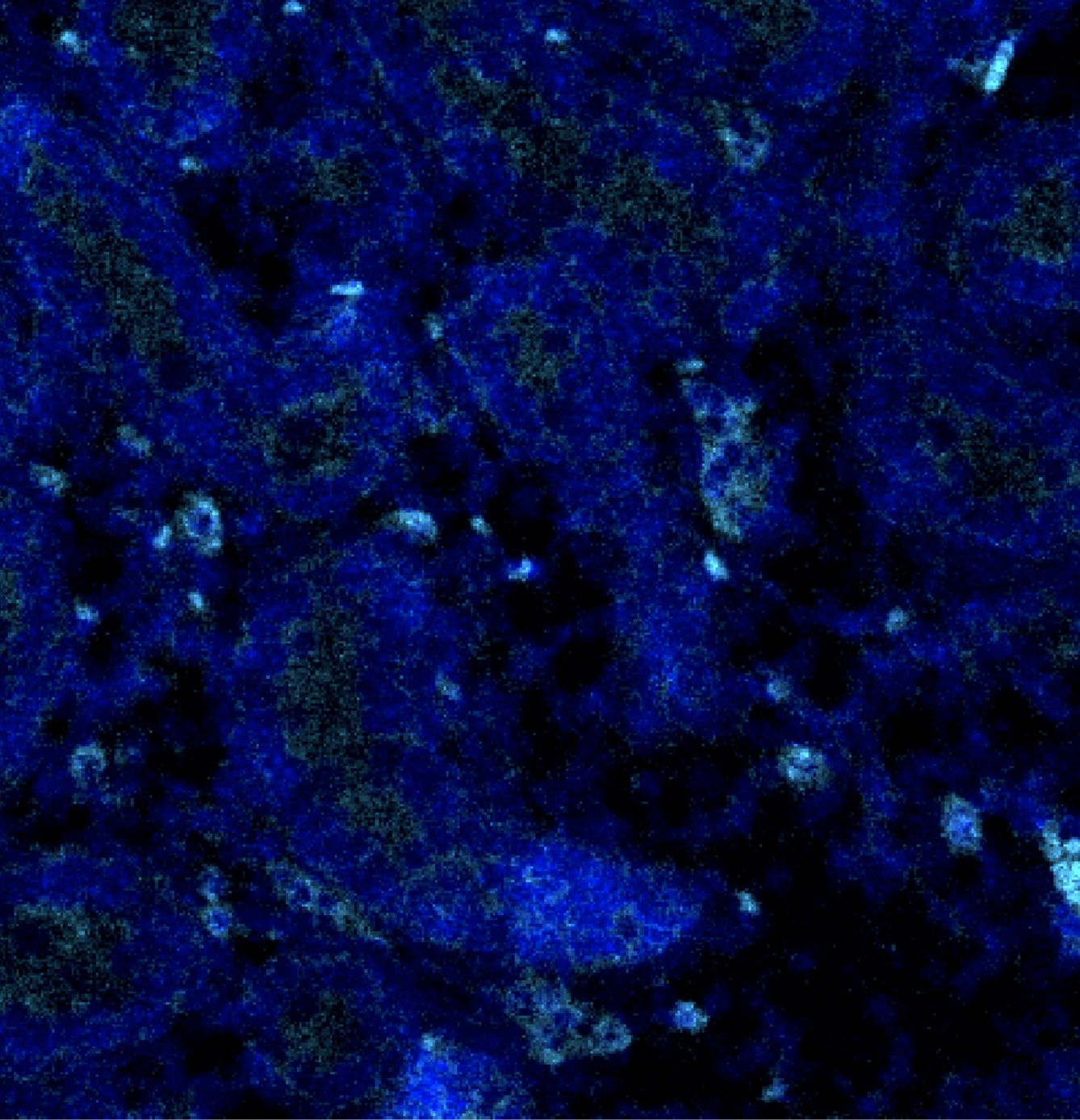

Supplement: Supplementary file 5 — Source Data Fig. 4 [file 44319_2023_31_MOESM5_ESM.zip › Figure 4/4C/8 Sf1_Het from Het.jpg]

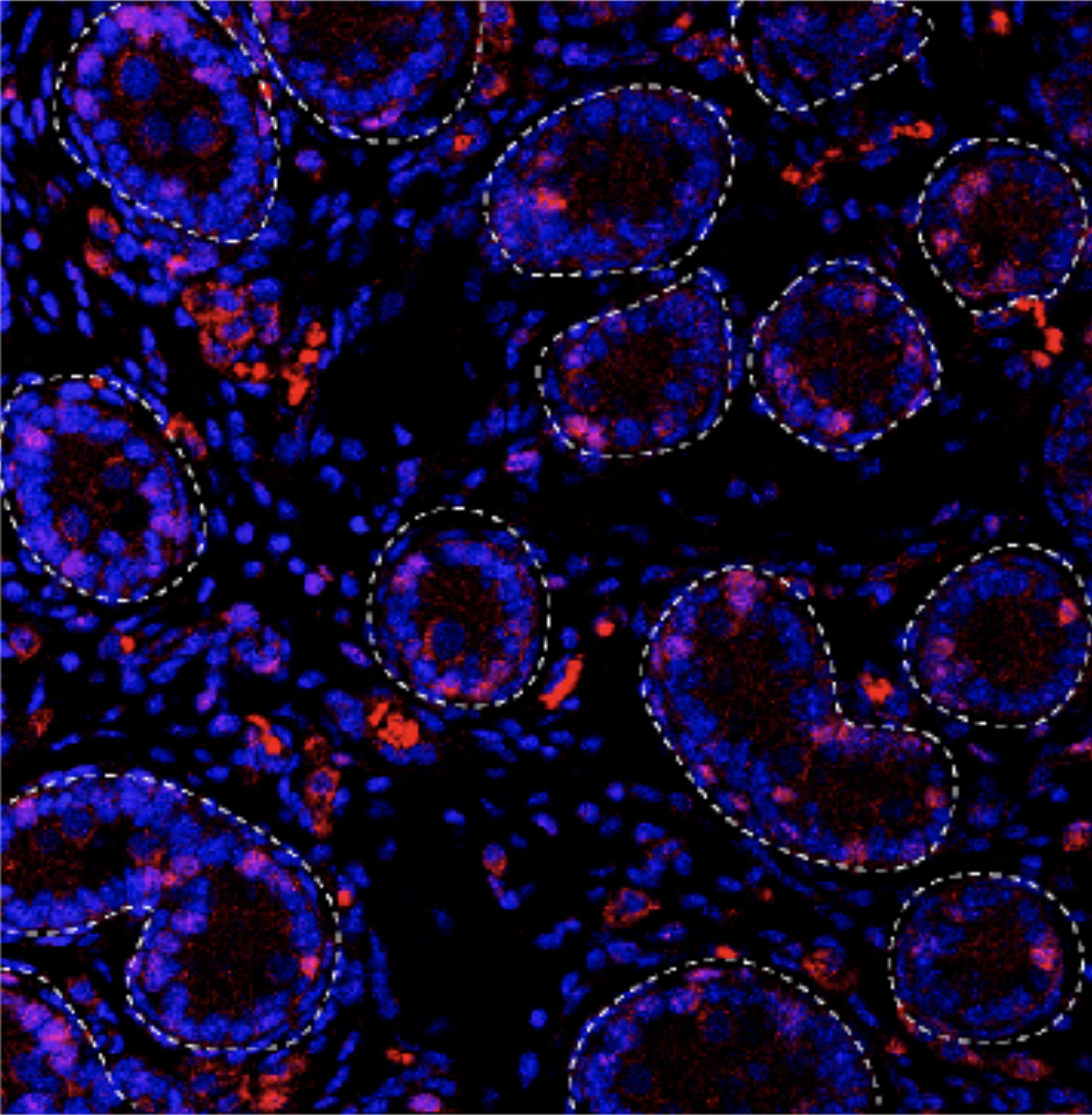

Supplement: Supplementary file 5 — Source Data Fig. 4 [file 44319_2023_31_MOESM5_ESM.zip › Figure 4/4C/1 BrdU_WT.jpg]

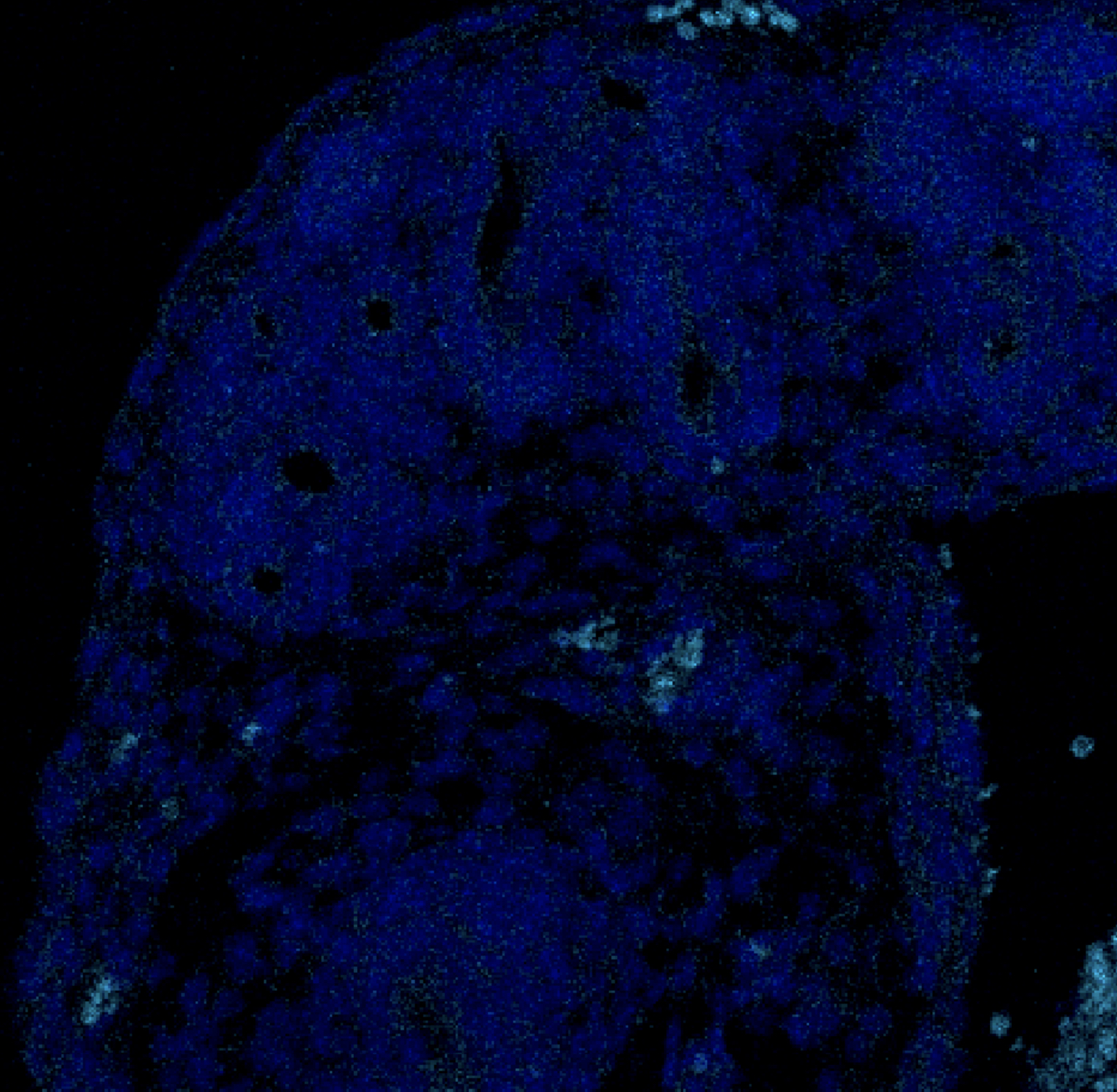

Supplement: Supplementary file 5 — Source Data Fig. 4 [file 44319_2023_31_MOESM5_ESM.zip › Figure 4/4C/11 Sf1_Homo from Het.jpg]

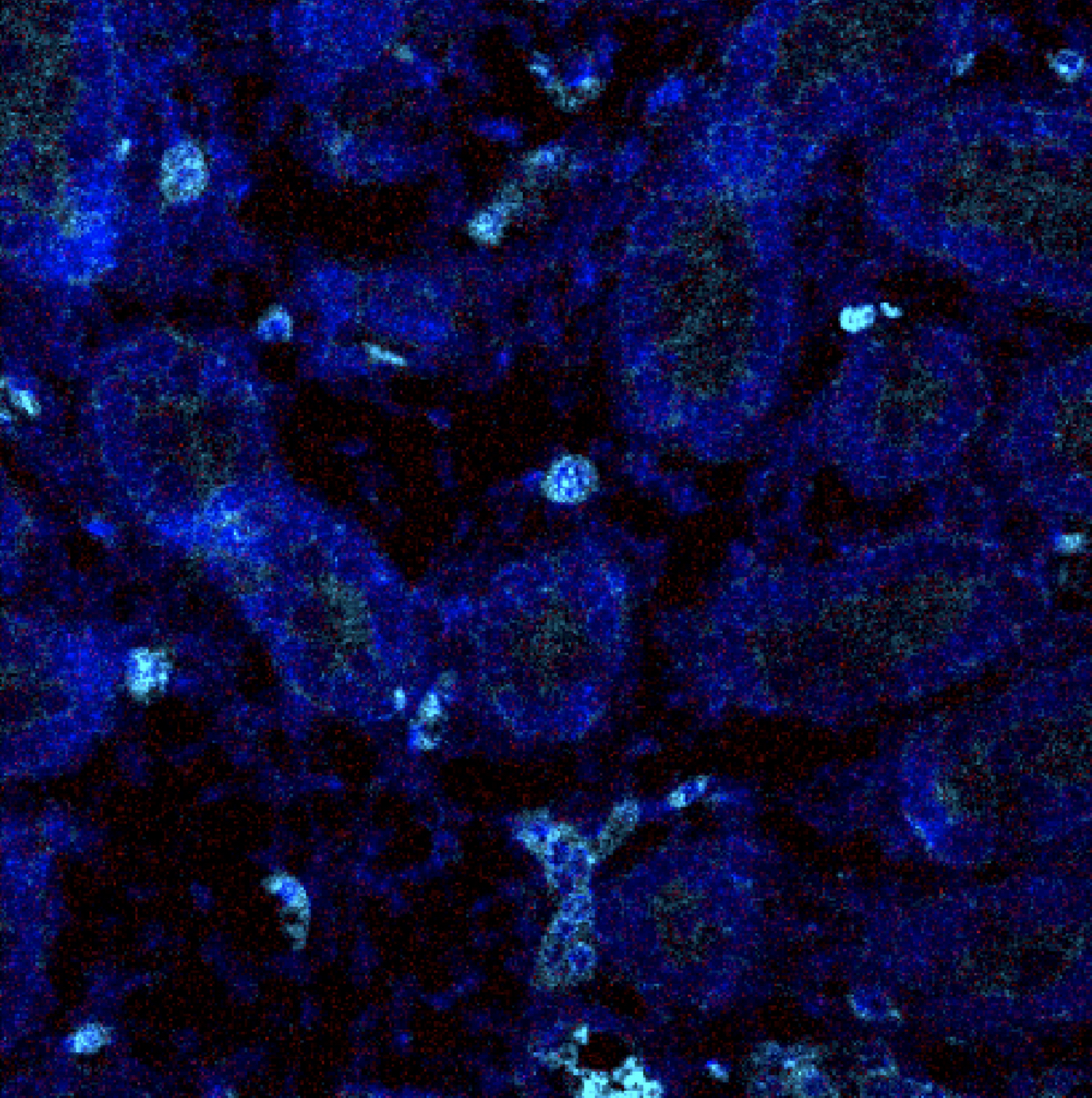

Supplement: Supplementary file 5 — Source Data Fig. 4 [file 44319_2023_31_MOESM5_ESM.zip › Figure 4/4C/7 Sf1_WT.jpg]

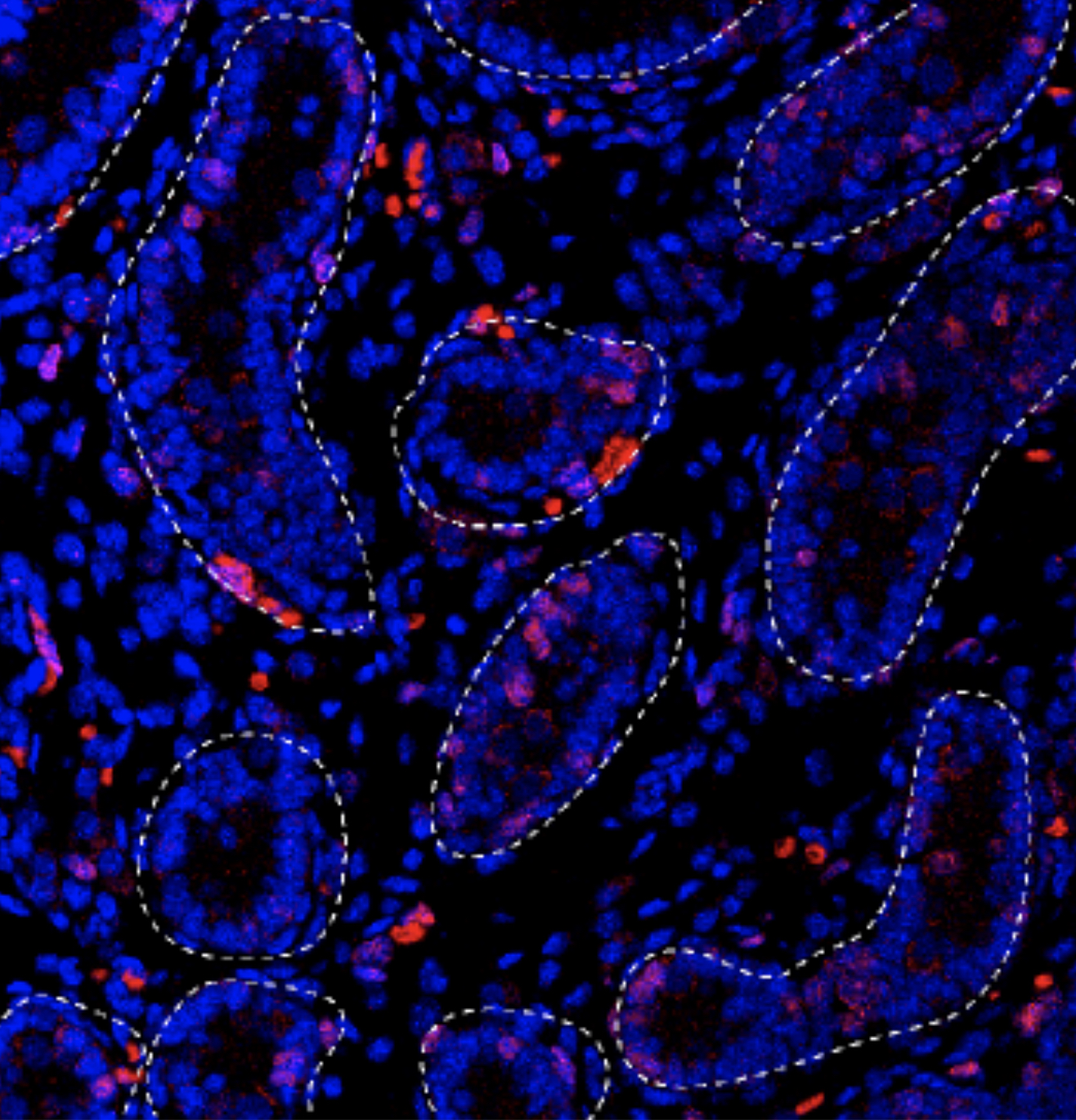

Supplement: Supplementary file 5 — Source Data Fig. 4 [file 44319_2023_31_MOESM5_ESM.zip › Figure 4/4C/4 BrdU_Het from Homo.jpg]

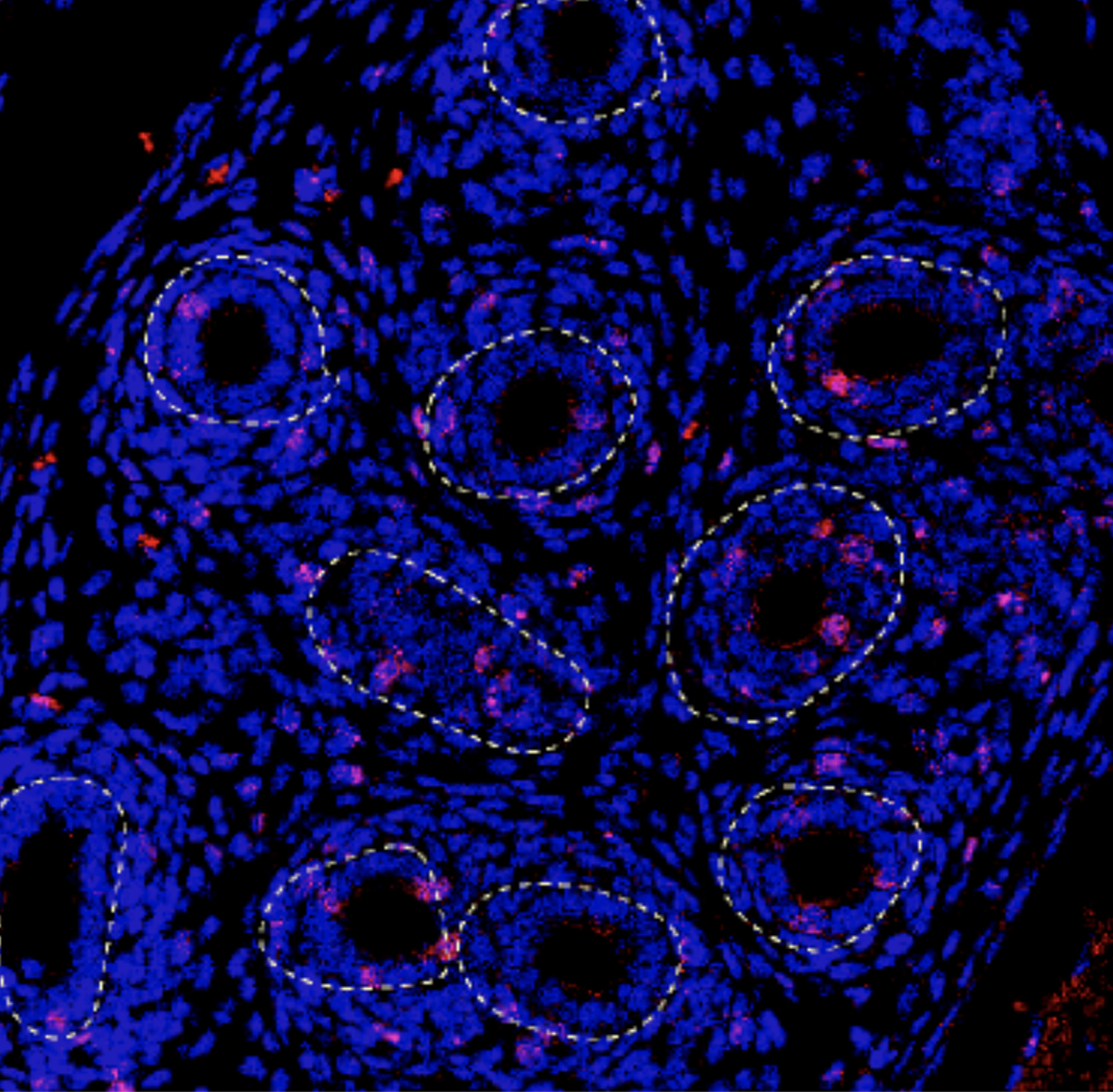

Supplement: Supplementary file 5 — Source Data Fig. 4 [file 44319_2023_31_MOESM5_ESM.zip › Figure 4/4C/5 BrdU_Homo from Het.jpg]

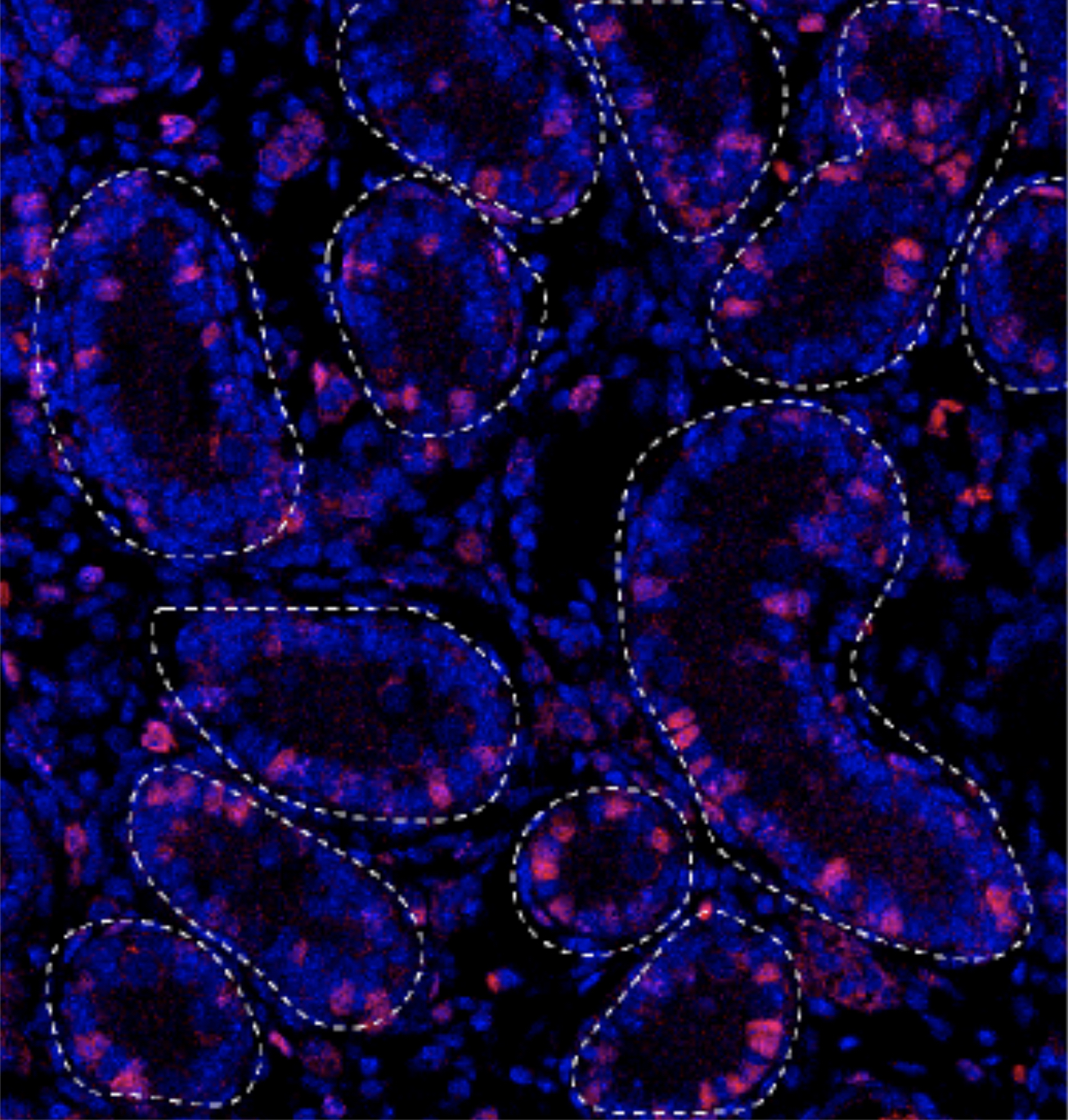

Supplement: Supplementary file 5 — Source Data Fig. 4 [file 44319_2023_31_MOESM5_ESM.zip › Figure 4/4C/2 BrdU_Het from Het.jpg]

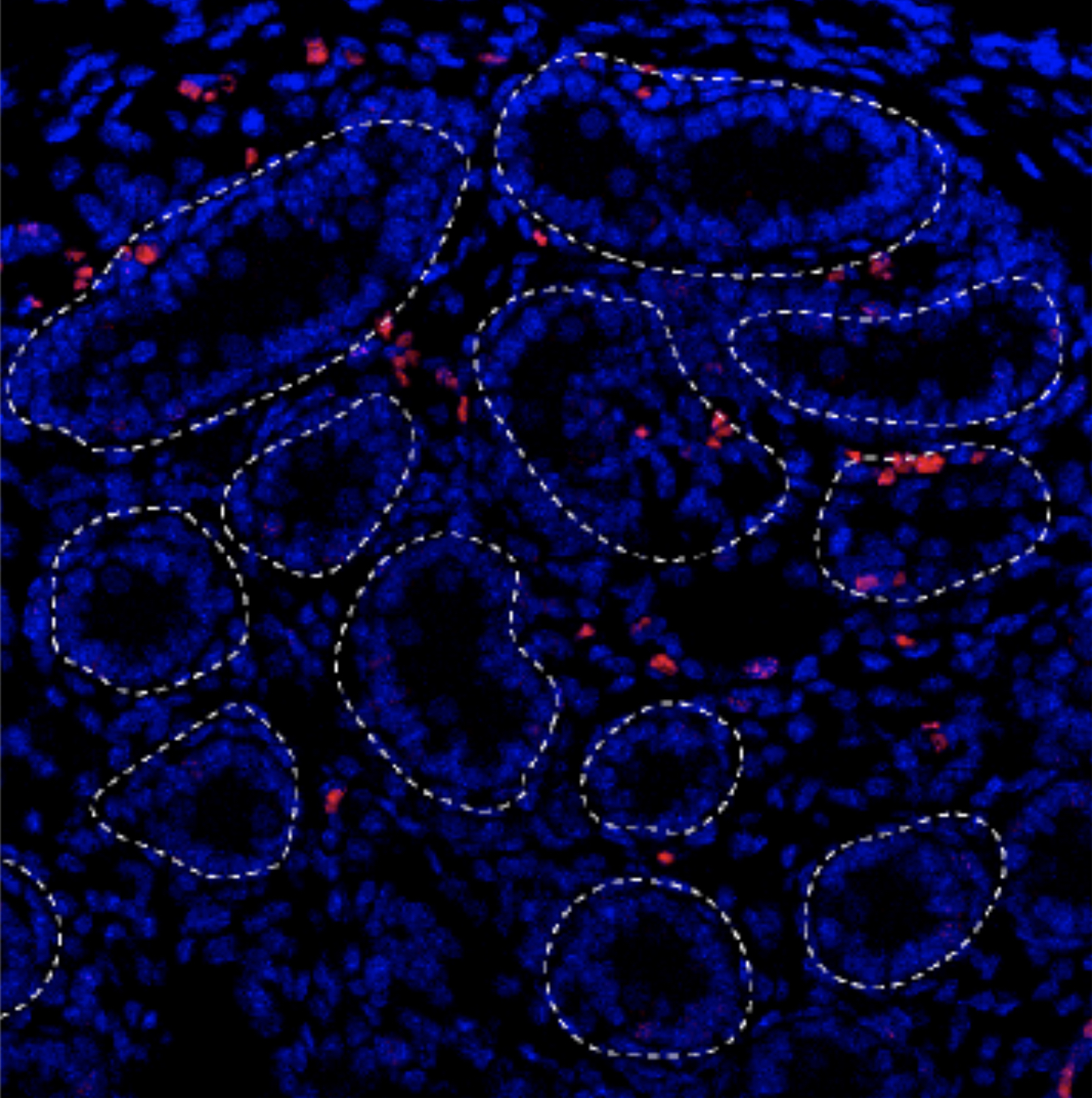

Supplement: Supplementary file 5 — Source Data Fig. 4 [file 44319_2023_31_MOESM5_ESM.zip › Figure 4/4C/6 BrdU_Homo from Homo.jpg]

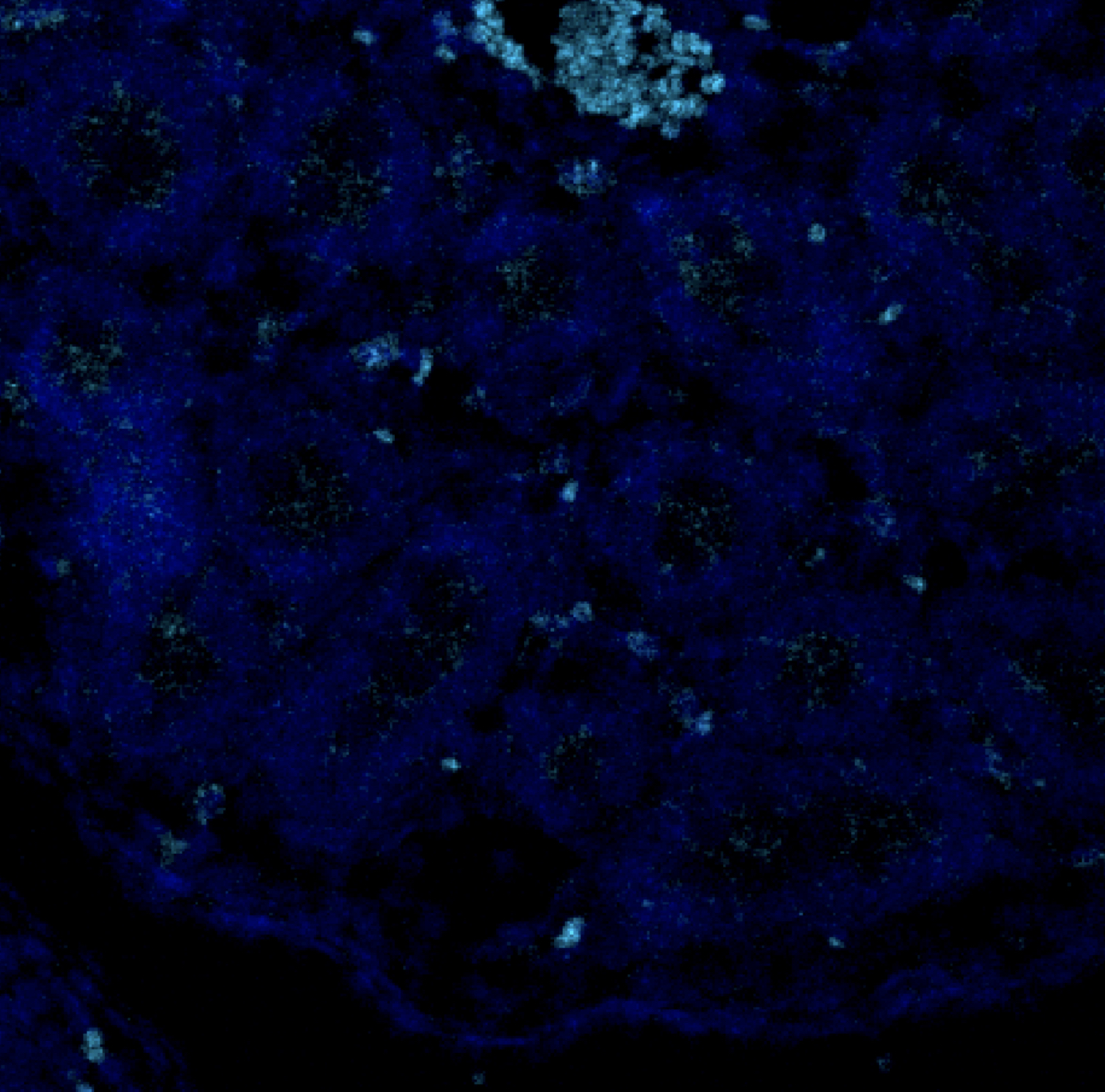

Supplement: Supplementary file 5 — Source Data Fig. 4 [file 44319_2023_31_MOESM5_ESM.zip › Figure 4/4C/12 Sf1_Homo from Homo.jpg]

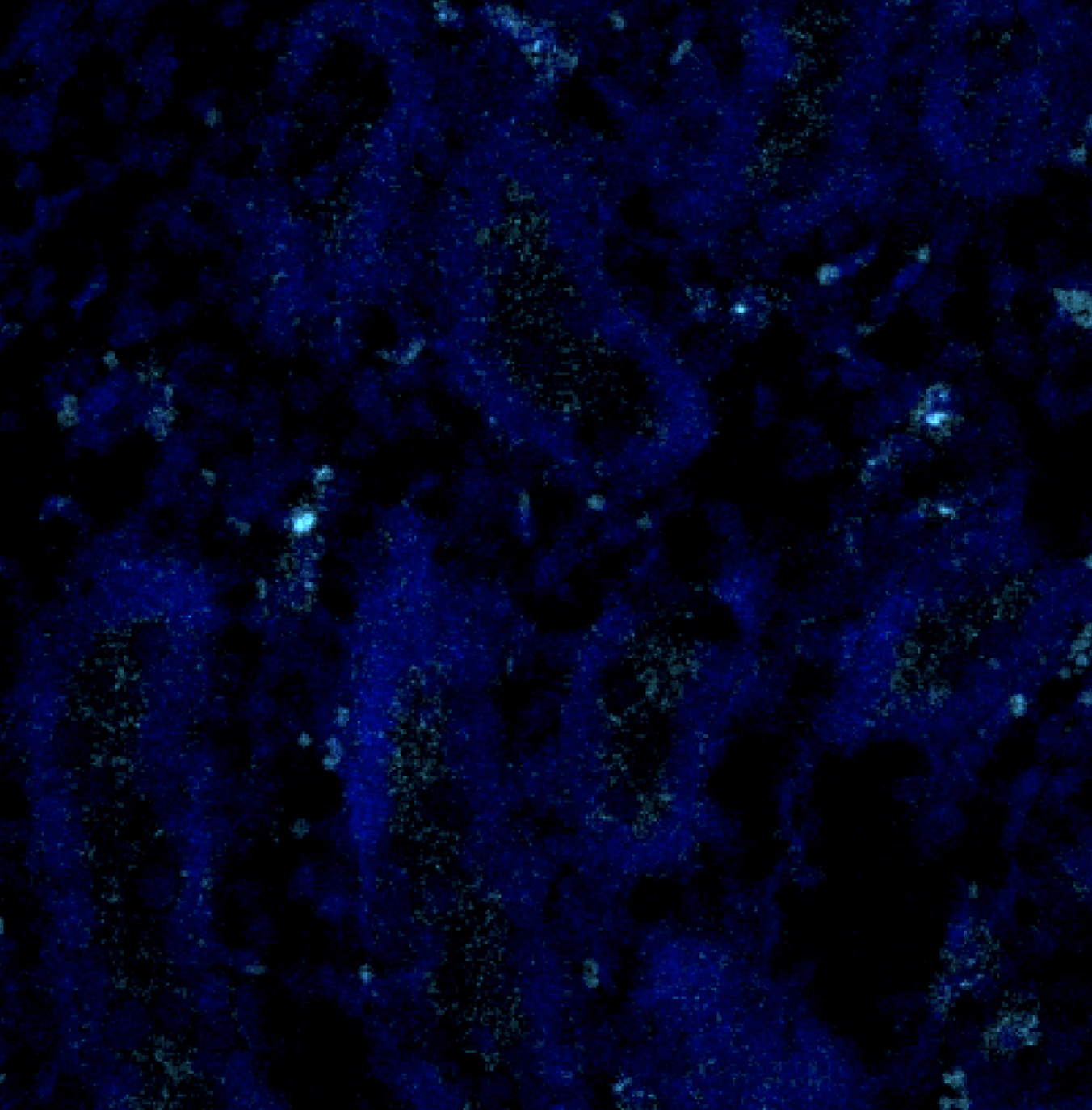

Supplement: Supplementary file 5 — Source Data Fig. 4 [file 44319_2023_31_MOESM5_ESM.zip › Figure 4/4C/10 Sf1_Het from Homo.jpg]

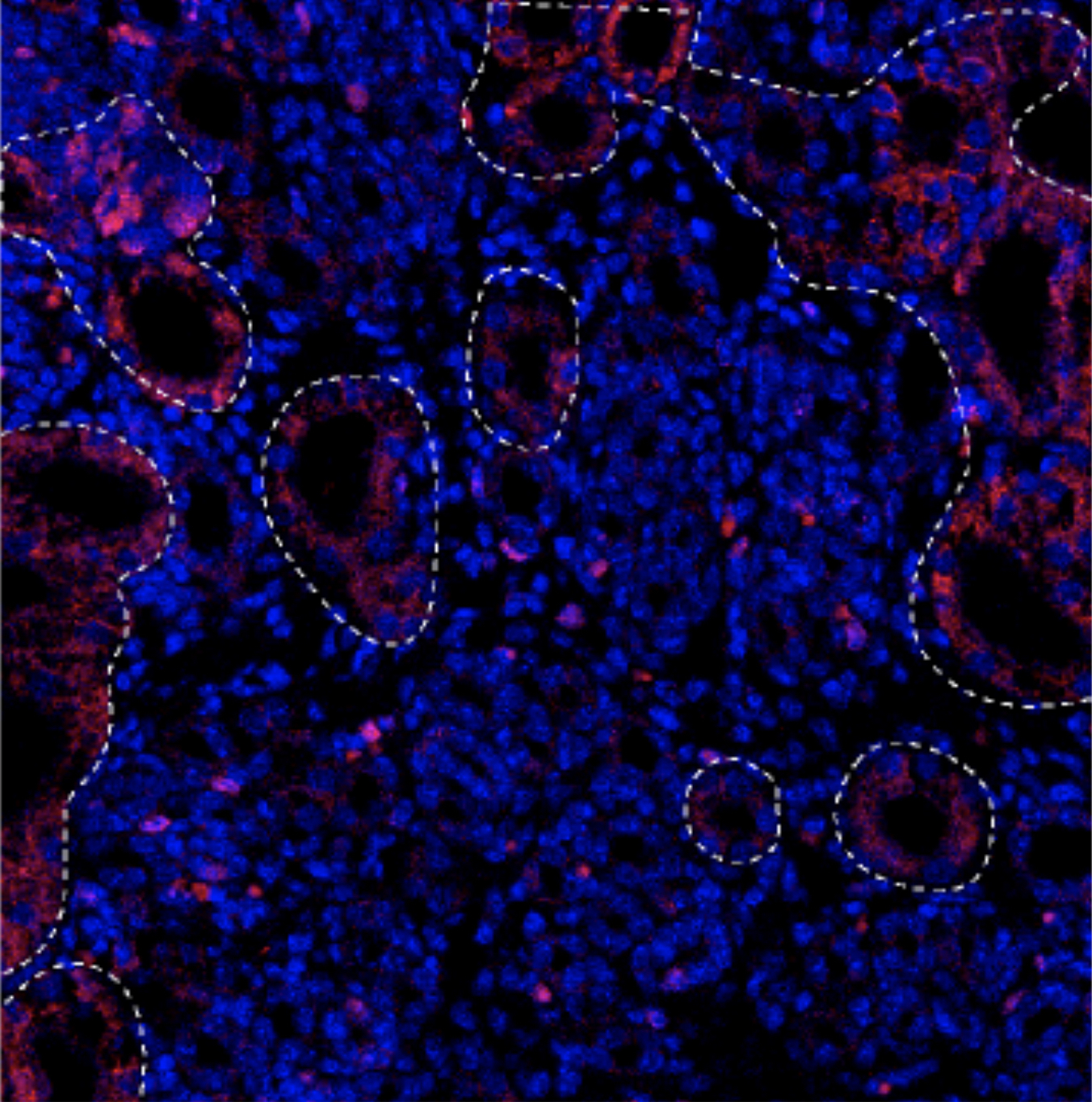

Supplement: Supplementary file 5 — Source Data Fig. 4 [file 44319_2023_31_MOESM5_ESM.zip › Figure 4/4C/3 BrdU_Het from WT.jpg]

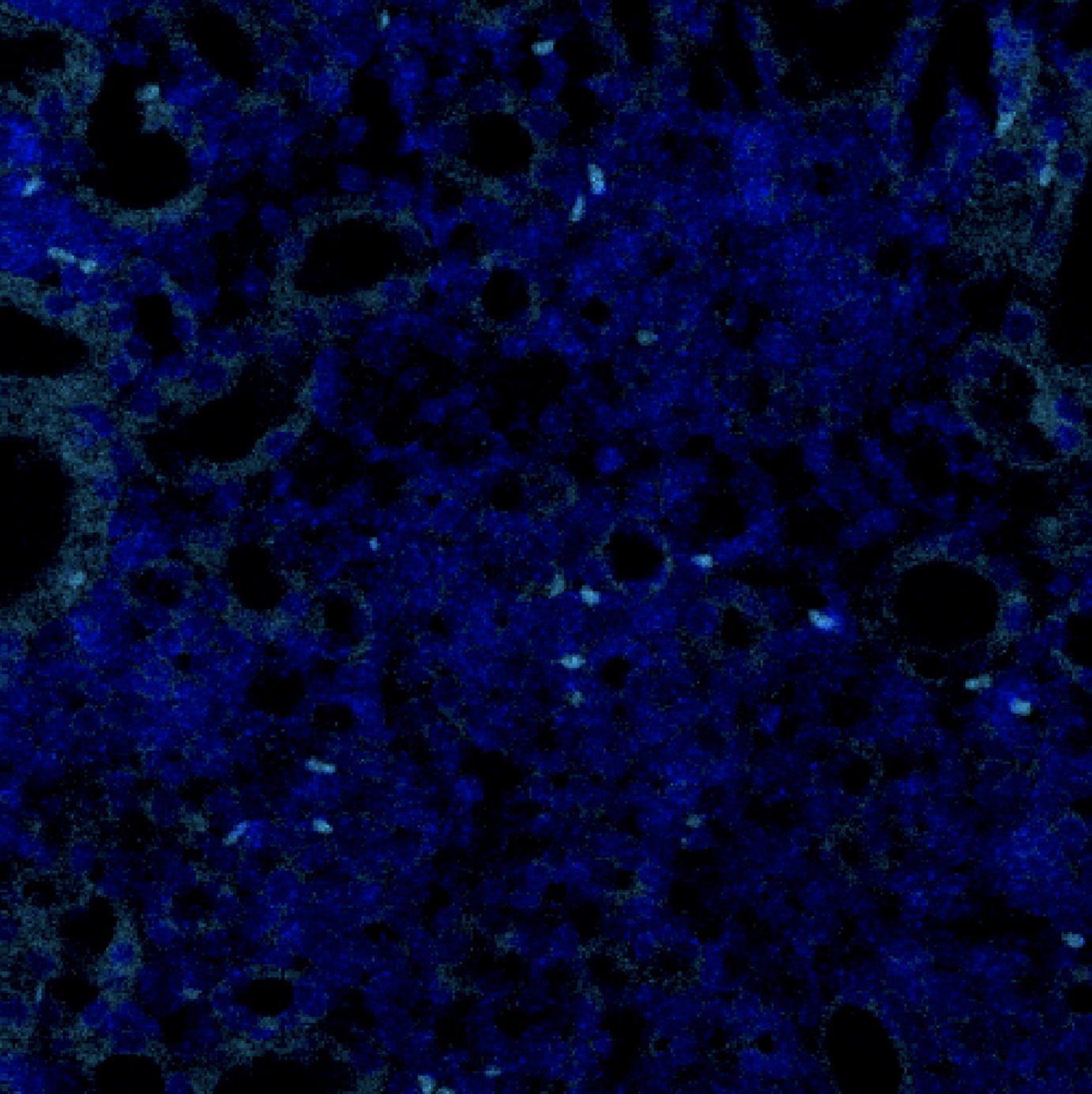

Supplement: Supplementary file 5 — Source Data Fig. 4 [file 44319_2023_31_MOESM5_ESM.zip › Figure 4/4C/9 Sf1_Het from WT.jpg]

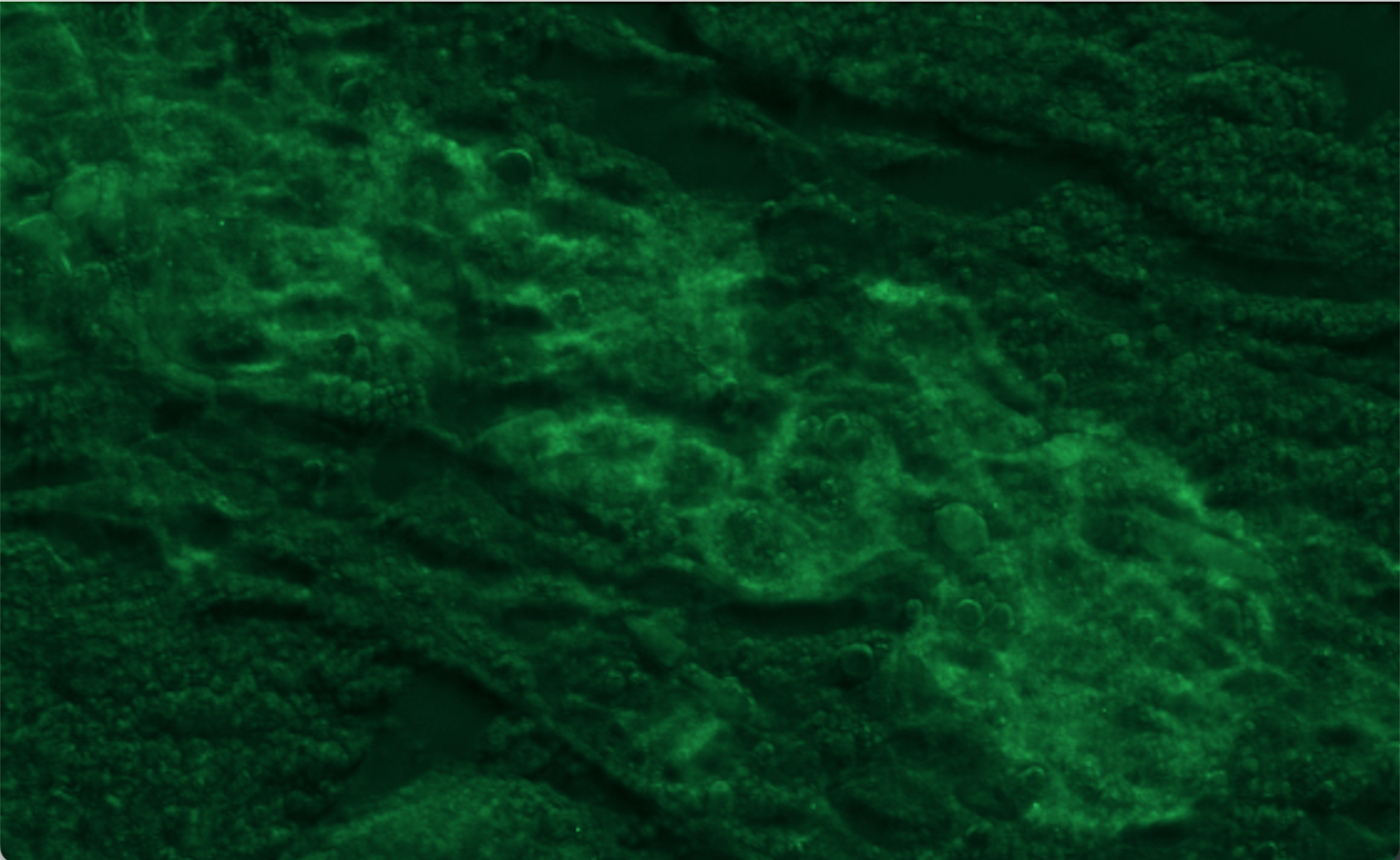

Supplement: Supplementary file 6 — Source Data Fig. 6 [file 44319_2023_31_MOESM6_ESM.zip › Figure 6/6E/1 PCK1_WT.tiff]

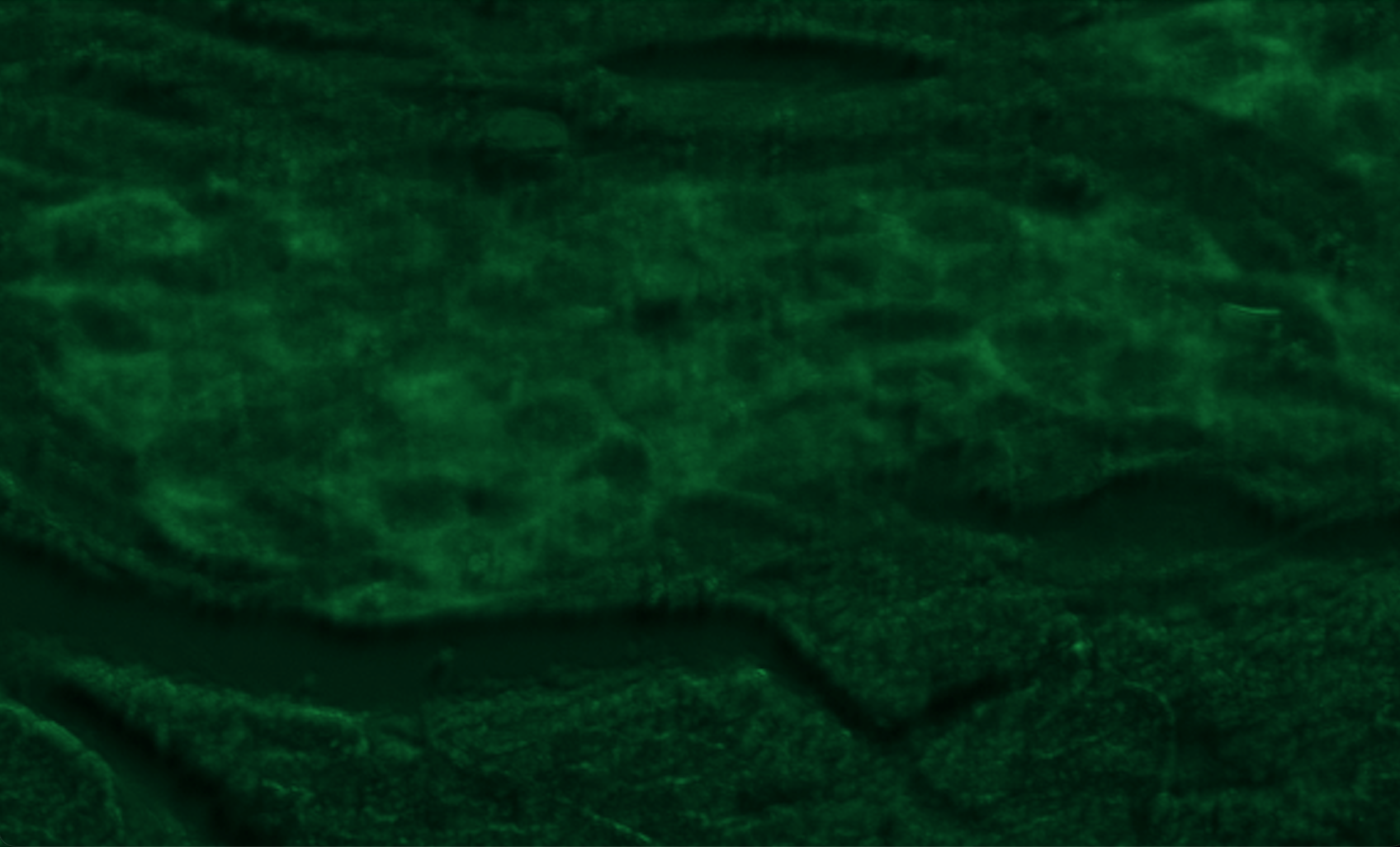

Supplement: Supplementary file 6 — Source Data Fig. 6 [file 44319_2023_31_MOESM6_ESM.zip › Figure 6/6E/2 PCK1_HET_FROM_HET.tiff]

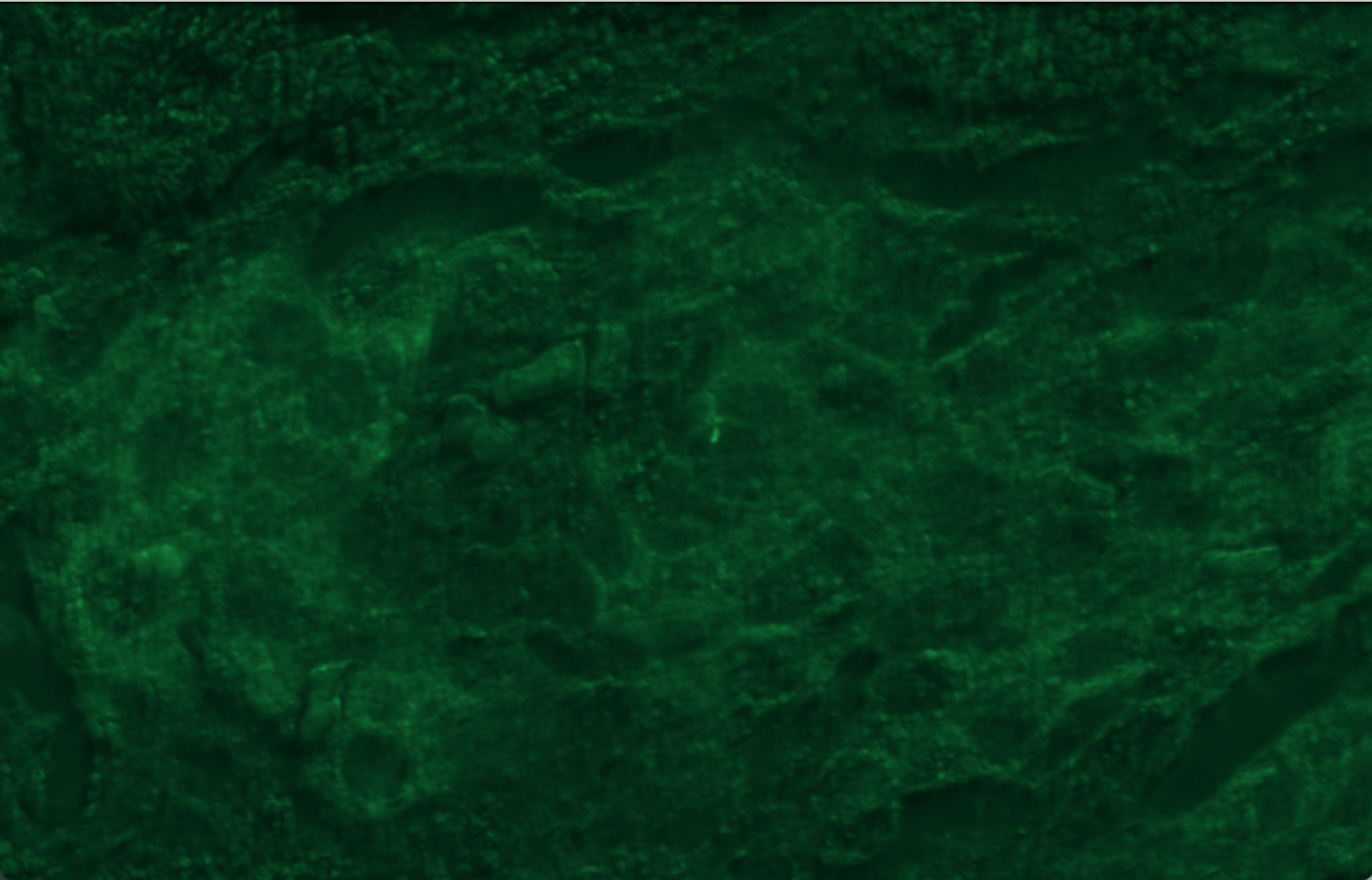

Supplement: Supplementary file 6 — Source Data Fig. 6 [file 44319_2023_31_MOESM6_ESM.zip › Figure 6/6E/3 PCK1_HET_FROM_KO.tiff]

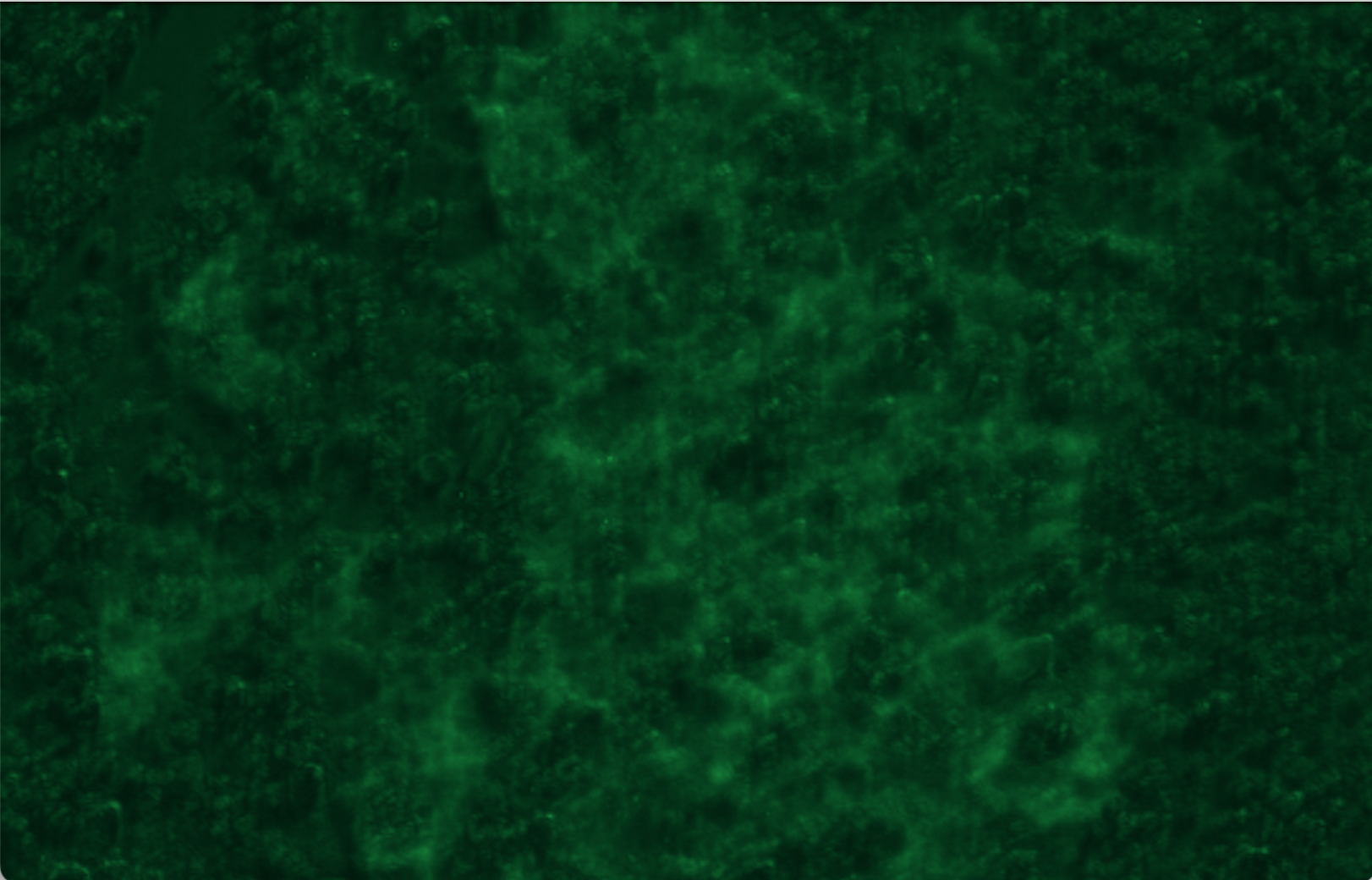

Supplement: Supplementary file 6 — Source Data Fig. 6 [file 44319_2023_31_MOESM6_ESM.zip › Figure 6/6E/5 PCK1_KO_FROM_KO.tiff]

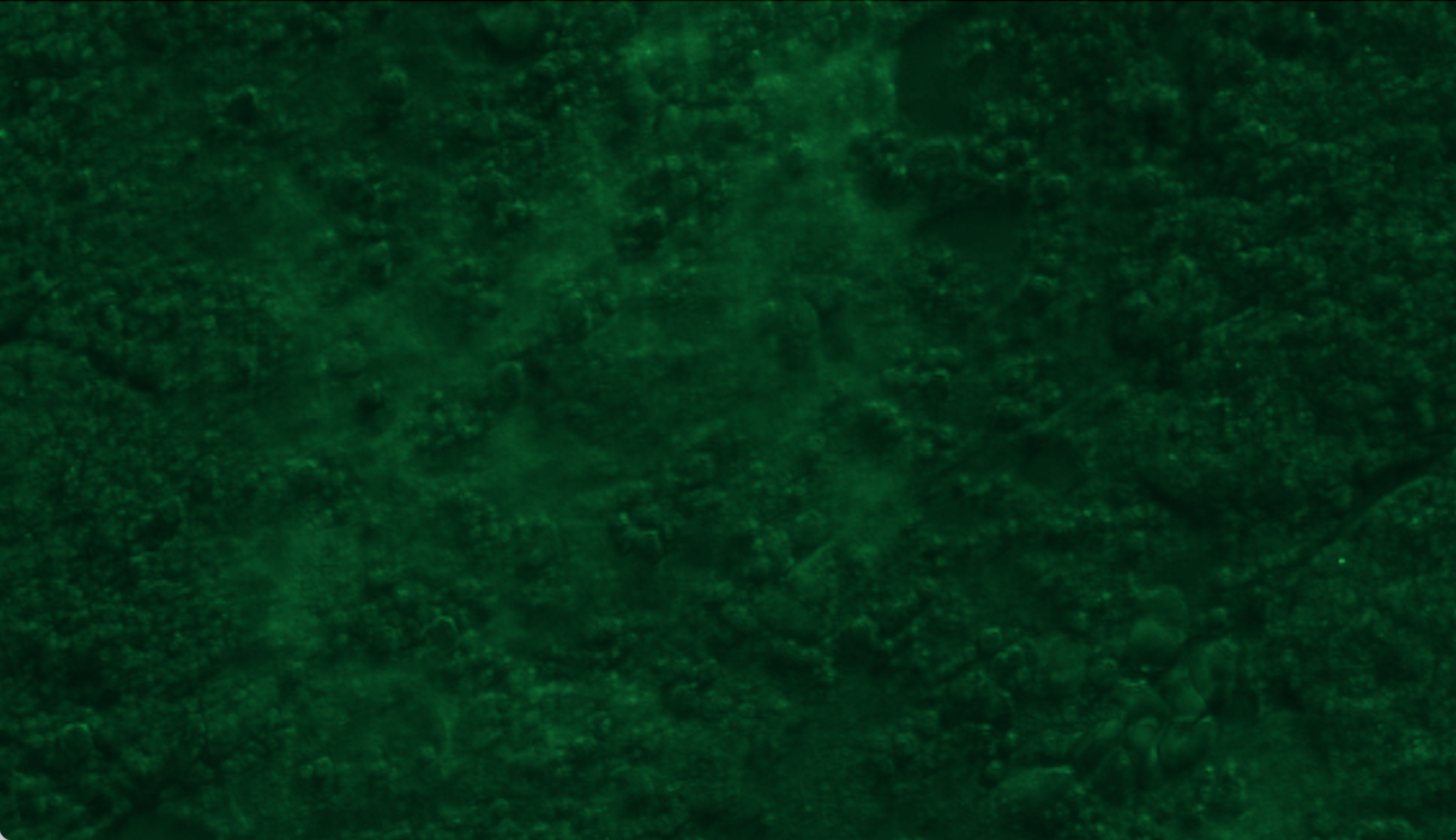

Supplement: Supplementary file 6 — Source Data Fig. 6 [file 44319_2023_31_MOESM6_ESM.zip › Figure 6/6E/4 PCK1_HET_FROM_WT.tiff]

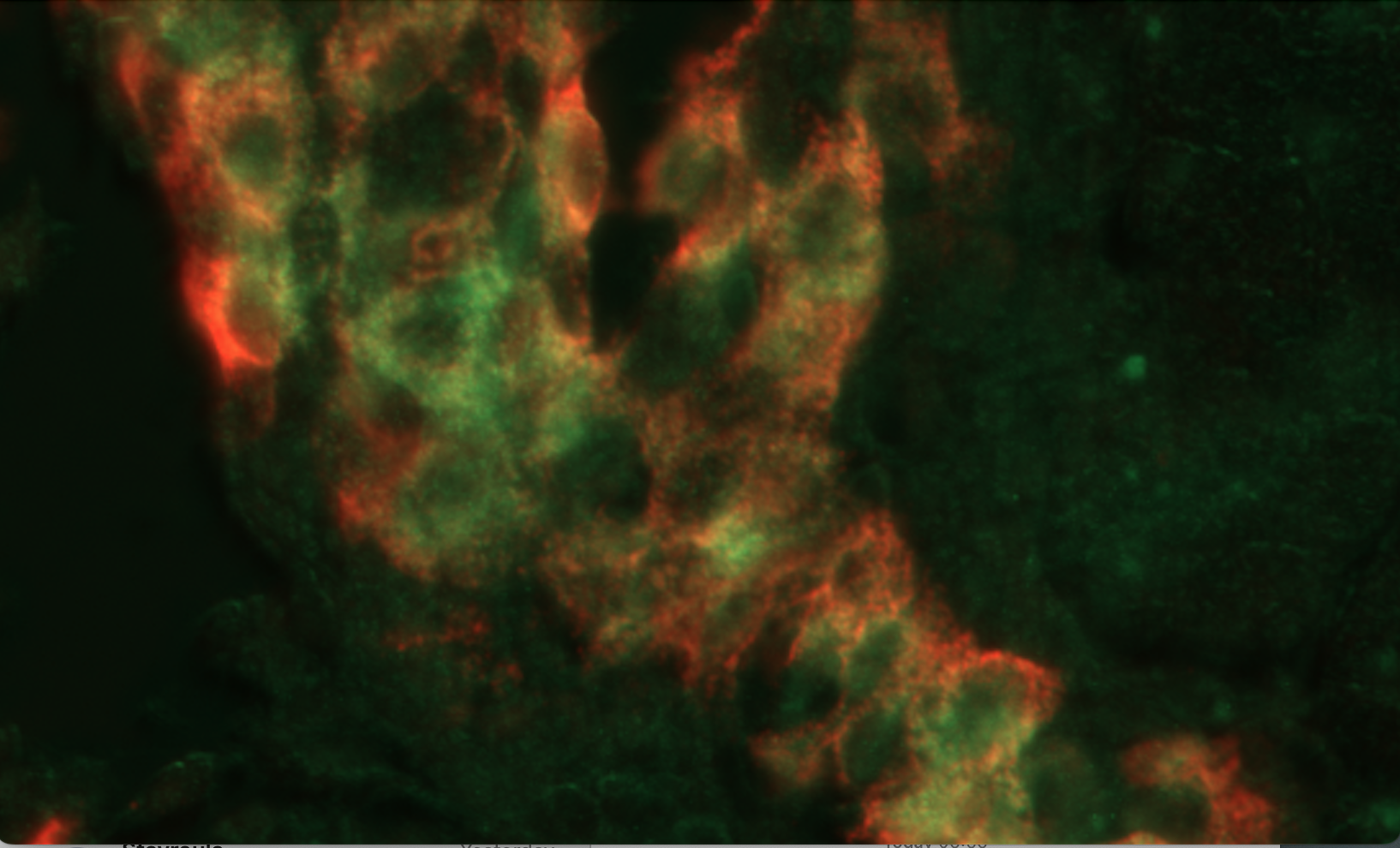

Supplement: Supplementary file 6 — Source Data Fig. 6 [file 44319_2023_31_MOESM6_ESM.zip › Figure 6/6D/1 CLOCK_WT.tiff]

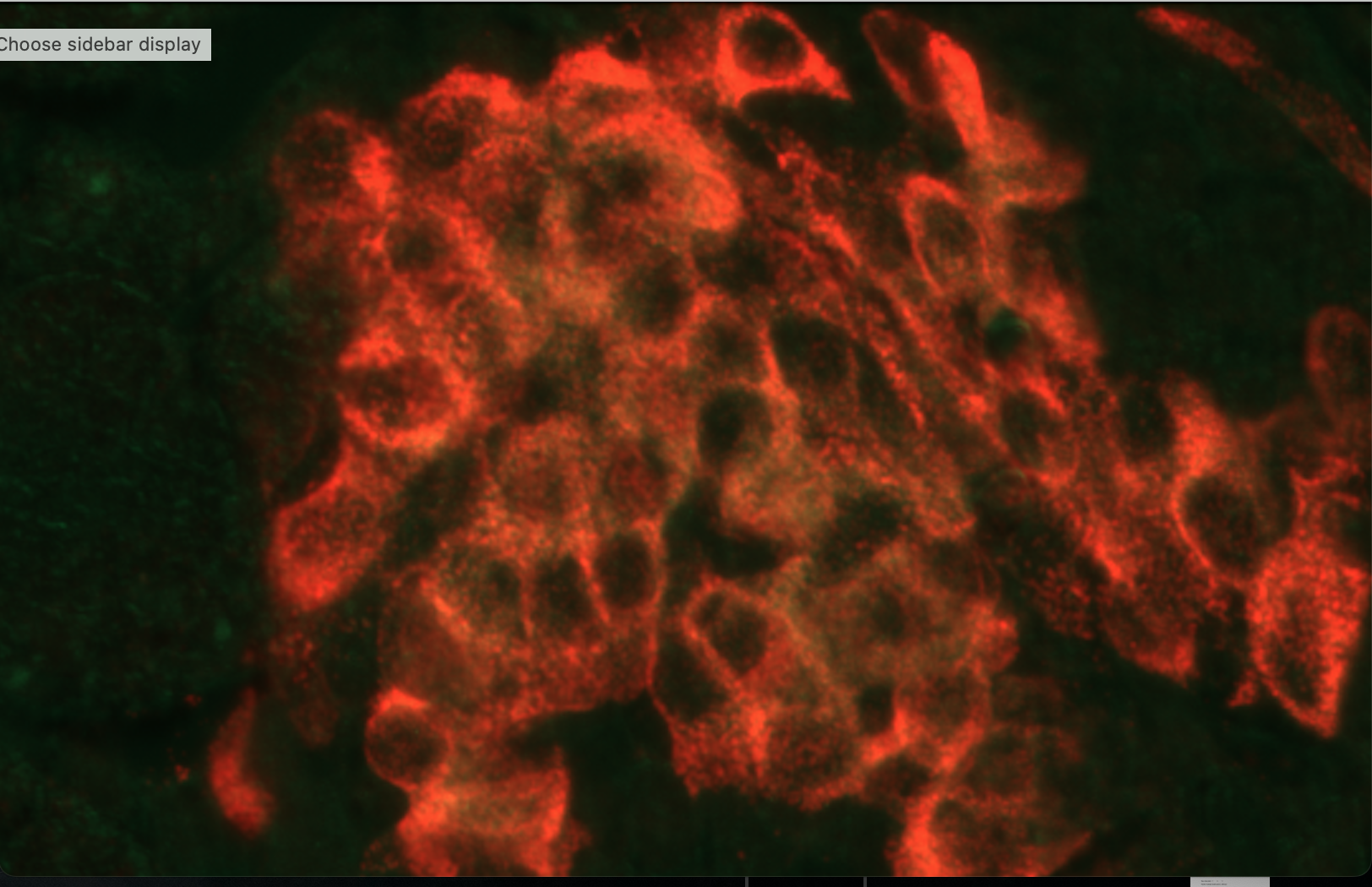

Supplement: Supplementary file 6 — Source Data Fig. 6 [file 44319_2023_31_MOESM6_ESM.zip › Figure 6/6D/3 CLOCK_HET_FROM_KO.tiff]

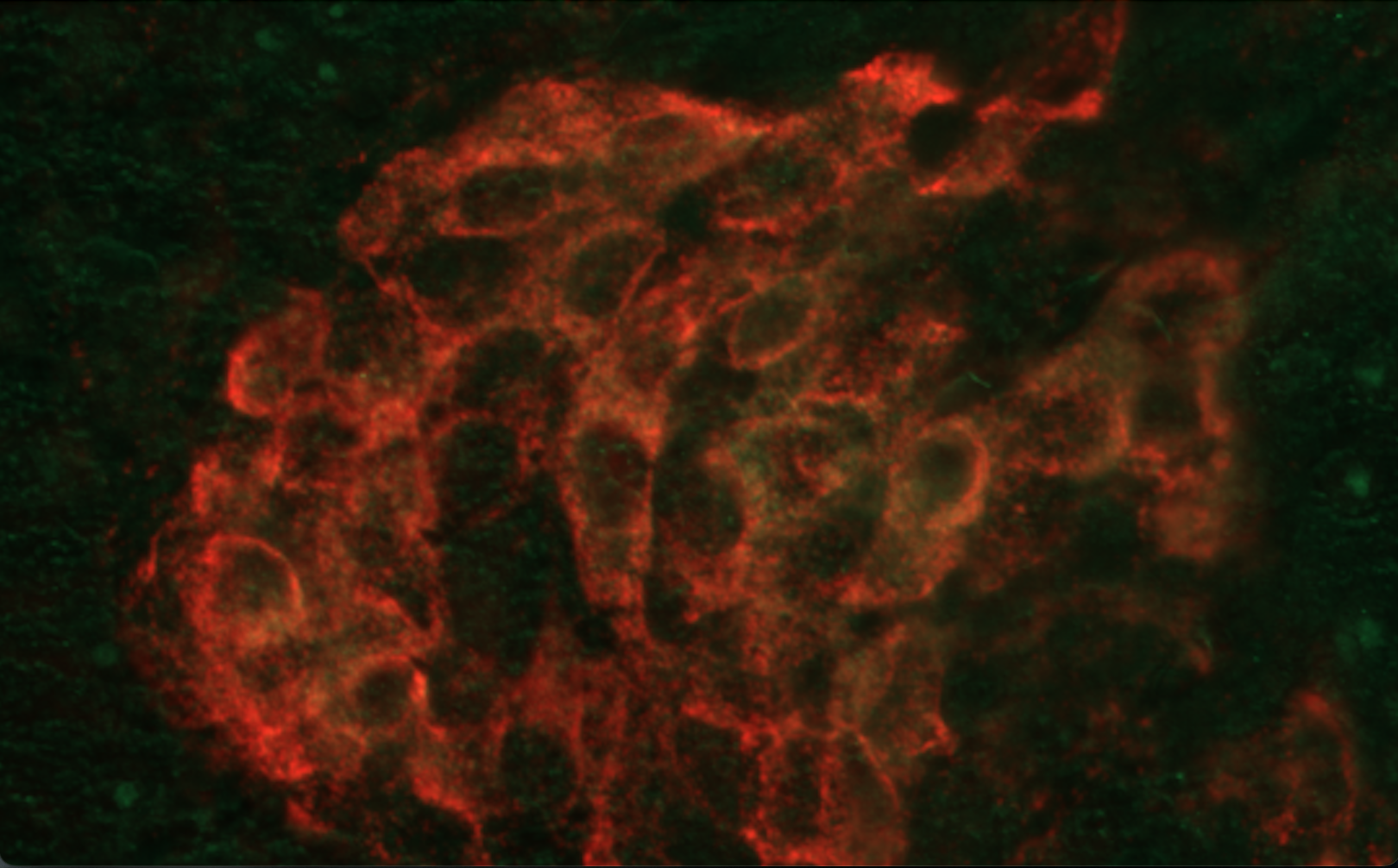

Supplement: Supplementary file 6 — Source Data Fig. 6 [file 44319_2023_31_MOESM6_ESM.zip › Figure 6/6D/5 CLOCK_KO_FROM_KO.tiff]

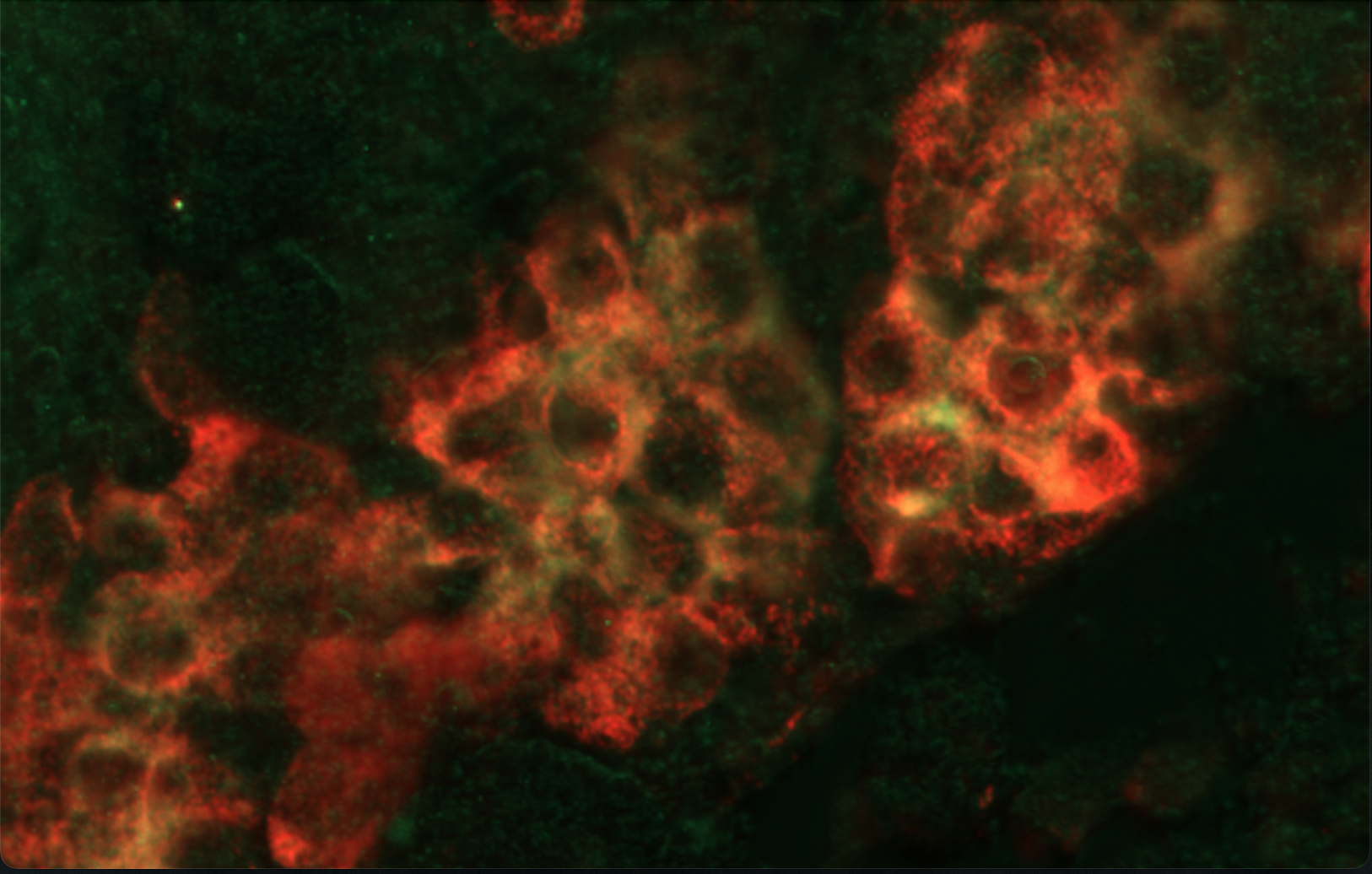

Supplement: Supplementary file 6 — Source Data Fig. 6 [file 44319_2023_31_MOESM6_ESM.zip › Figure 6/6D/2 CLOCK_HET_FROM_HET.tiff]

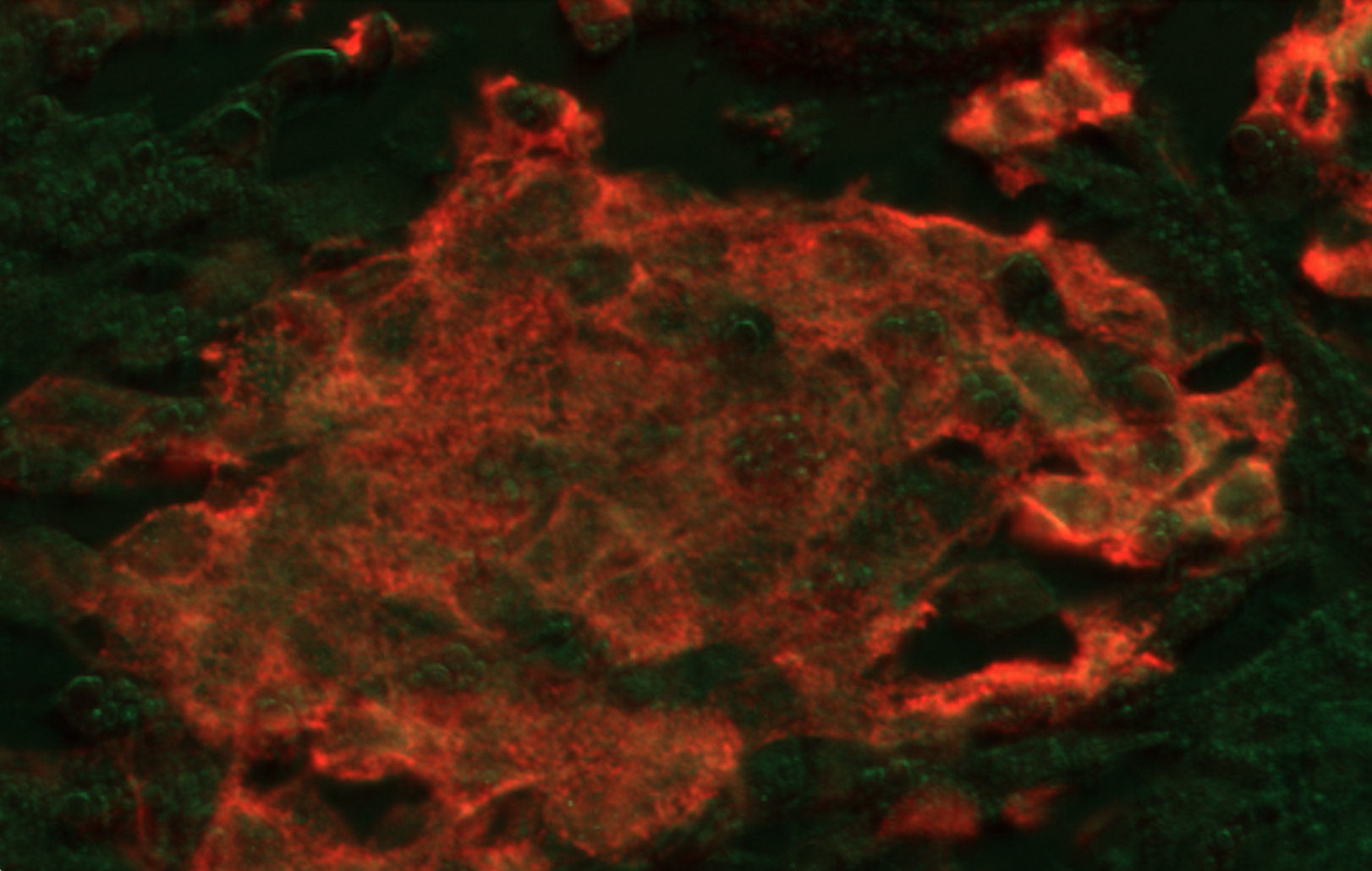

Supplement: Supplementary file 6 — Source Data Fig. 6 [file 44319_2023_31_MOESM6_ESM.zip › Figure 6/6D/4 CLOCK_HET_FROM_WT.tiff]

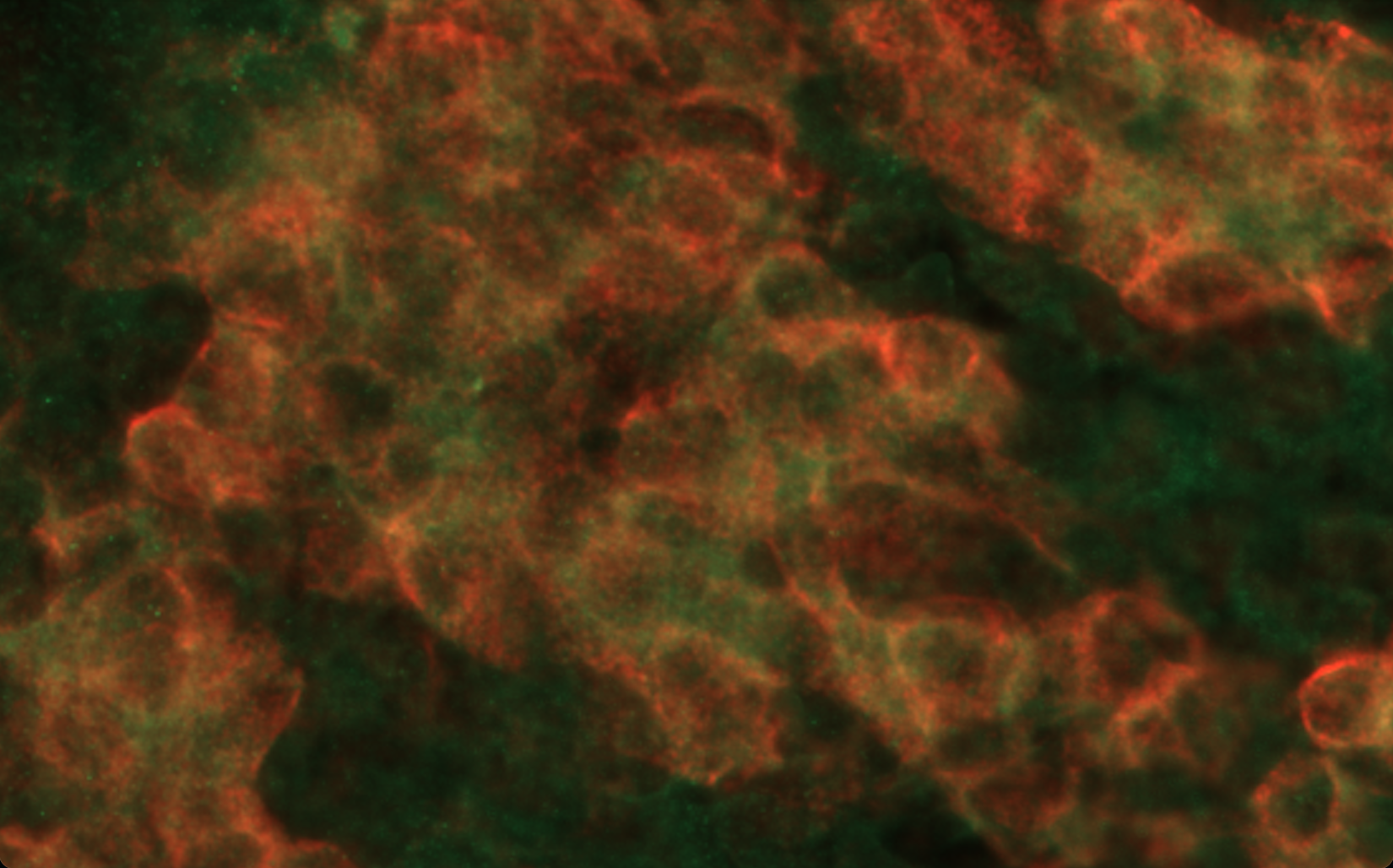

Supplement: Supplementary file 6 — Source Data Fig. 6 [file 44319_2023_31_MOESM6_ESM.zip › Figure 6/6C/3 BMAL_HET_FROM_KO.tiff]

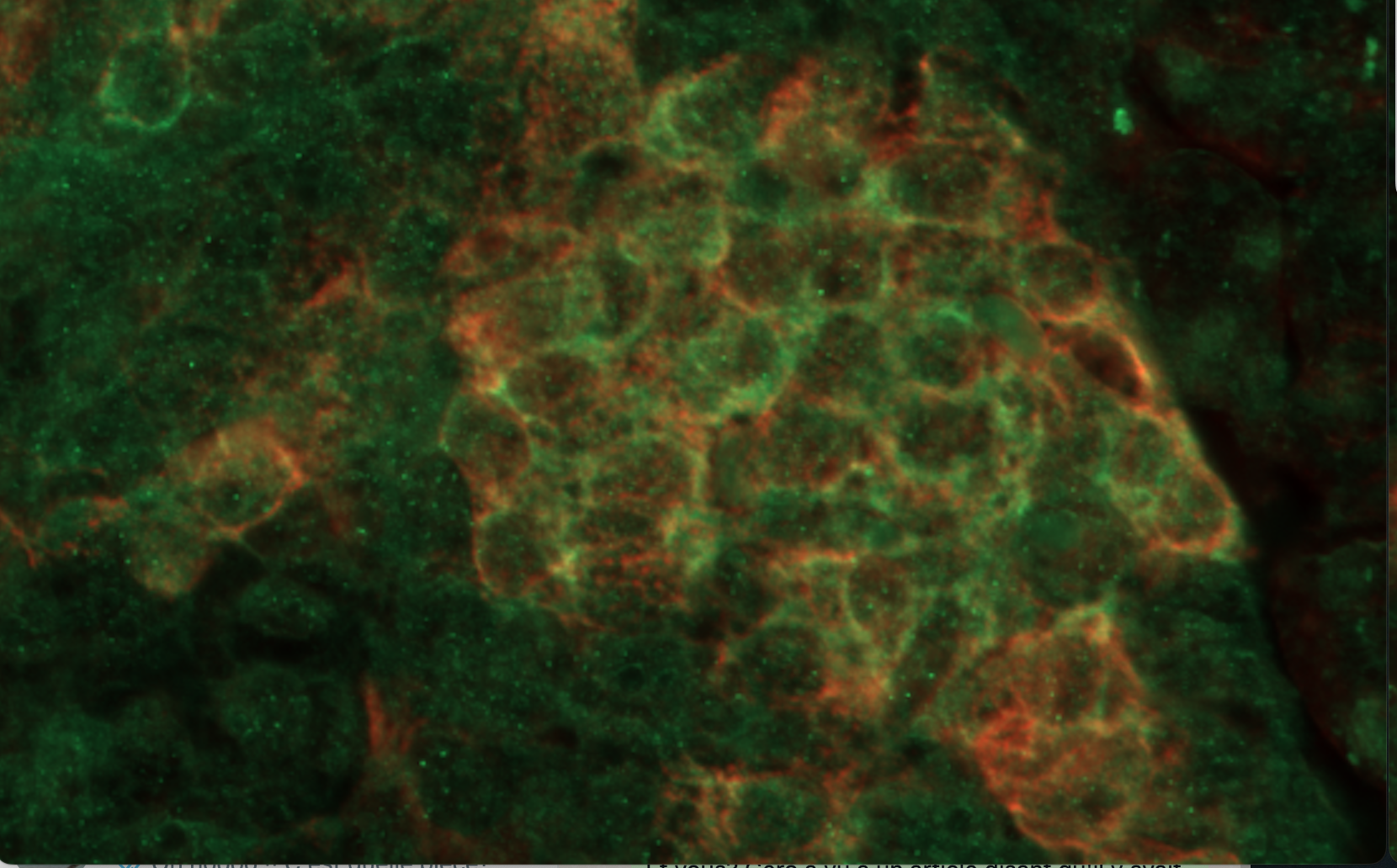

Supplement: Supplementary file 6 — Source Data Fig. 6 [file 44319_2023_31_MOESM6_ESM.zip › Figure 6/6C/4 BMAL_HET_FROM_WT.tiff]

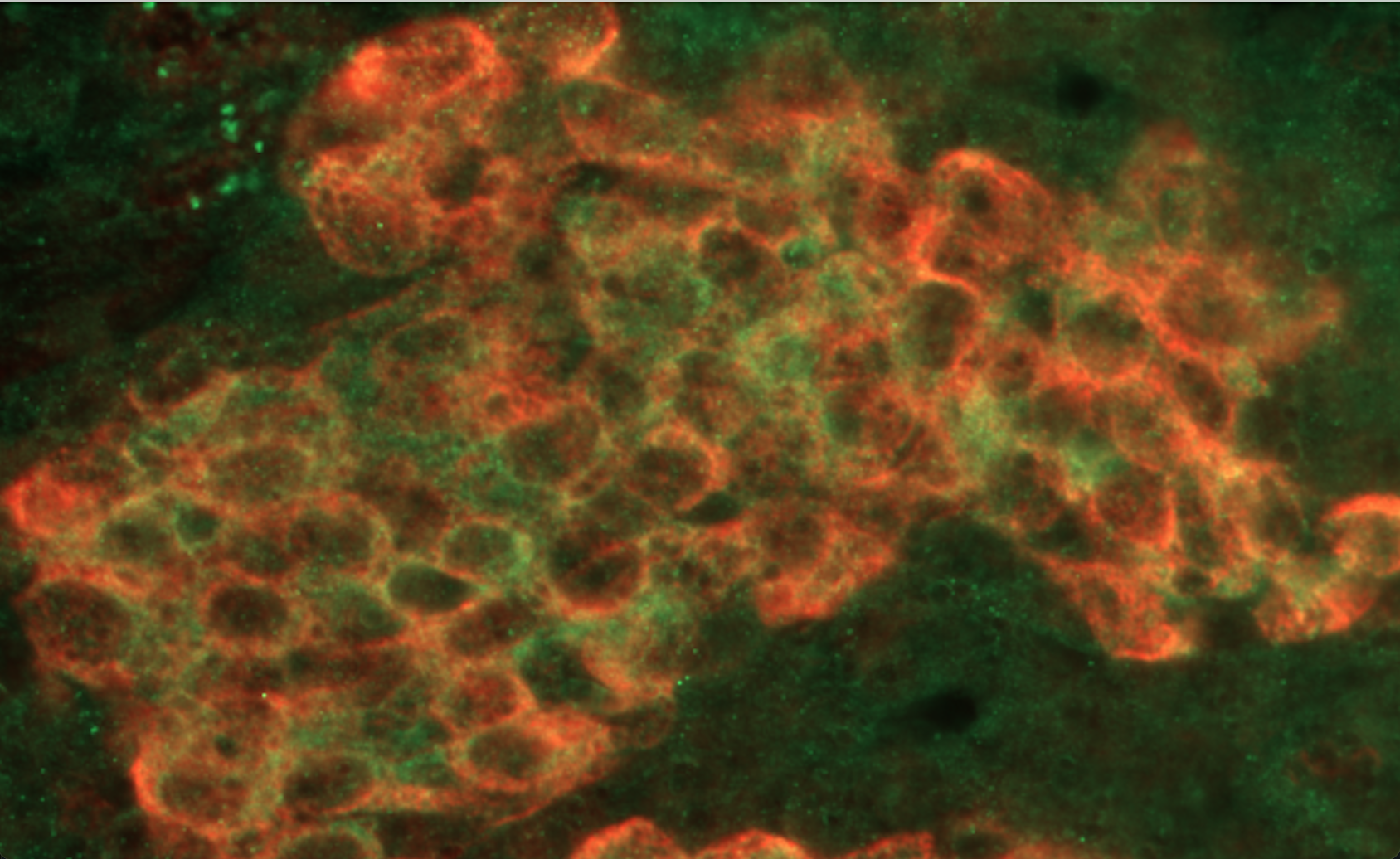

Supplement: Supplementary file 6 — Source Data Fig. 6 [file 44319_2023_31_MOESM6_ESM.zip › Figure 6/6C/2 BMAL_HET_FROM_HET.tiff]

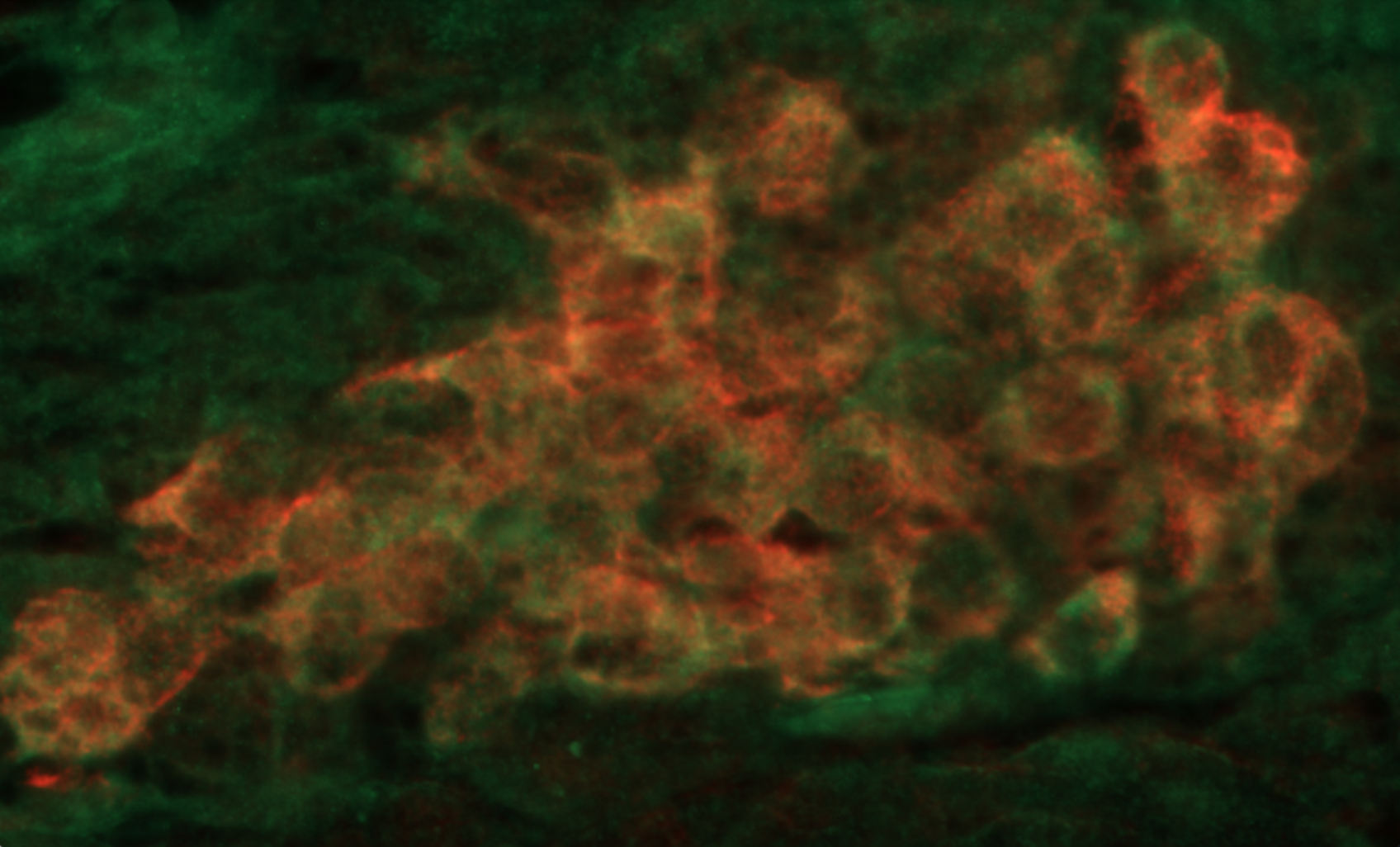

Supplement: Supplementary file 6 — Source Data Fig. 6 [file 44319_2023_31_MOESM6_ESM.zip › Figure 6/6C/5 BMAL_KO_FROM_KO.tiff]

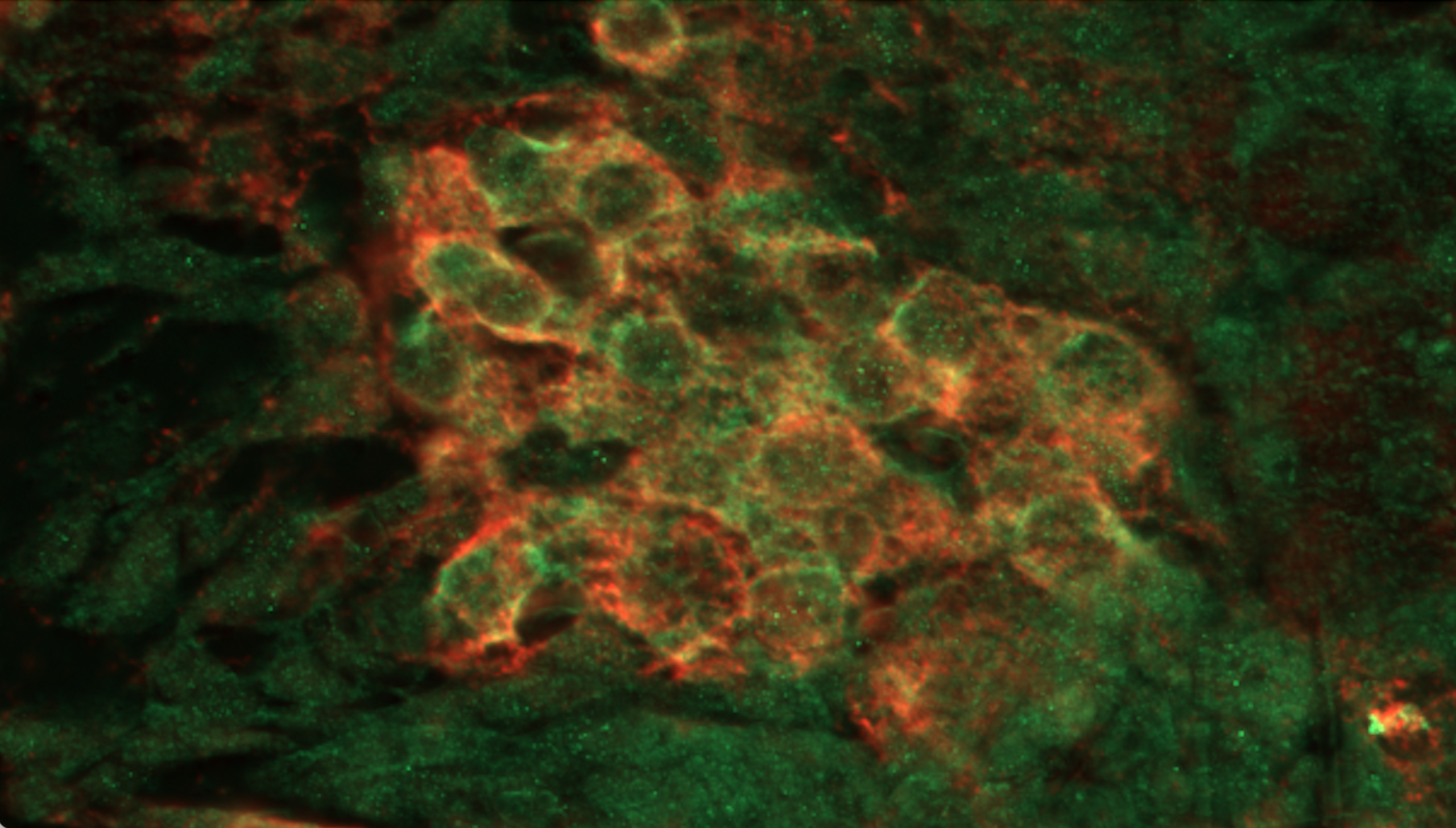

Supplement: Supplementary file 6 — Source Data Fig. 6 [file 44319_2023_31_MOESM6_ESM.zip › Figure 6/6C/1 BMAL_WT.tiff]
