# Supplementary material for: Osteocalcin of maternal and embryonic origins synergize to establish homeostasis in offspring
Source: EMBO Rep. 2024 Jan 16;25(2):12. doi: 10.1038/s44319-023-00031-3 (PMC10897216; doi:10.1038/s44319-023-00031-3)
Supplement: Supplementary file 8 — Expanded View Figures [file 44319_2023_31_MOESM8_ESM.pdf]

Expanded View Figures

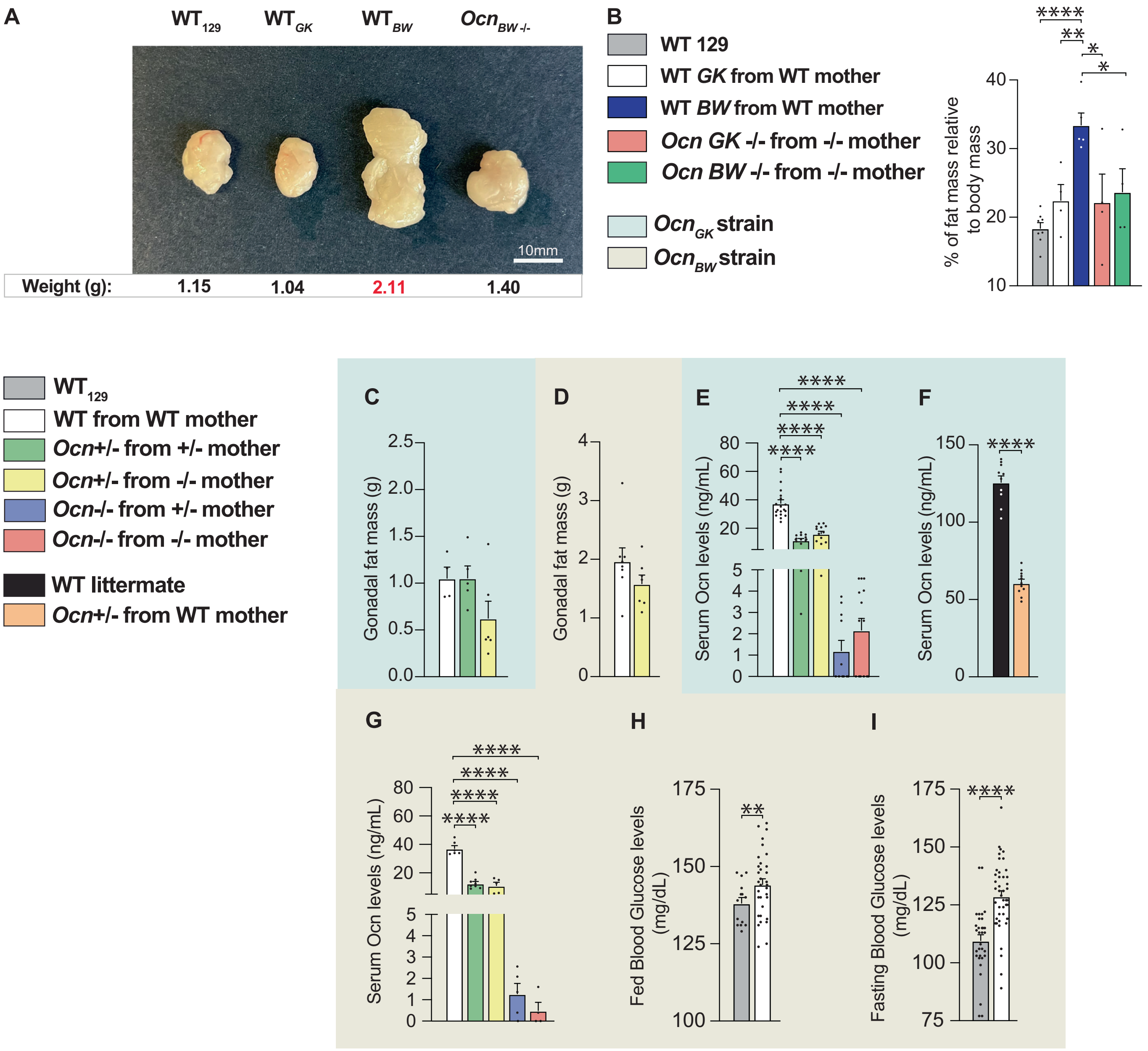

Figure EV1. Characterization of three mouse models of *Ocn* deletion.

(**A**) Photomicrographs of gonadal fat pads from WT_{129}, WT_{BW}, and $Ocn_{BW}-/-$ mice. Weight for each group is shown below each representative fat pad image. (**B**) Percent fat mass relative to body weight in WT_{129}, WT_{BW}, and $Ocn_{BW}-/-$ mice assessed by EchoMRI. $n = 4$ or more mice per genotype analyzed. (**C, D**) Gonadal fat mass in different genotypes of 12 weeks-old Ocn_{GK} mice (**C**). $n = 4$ or more mice per genotype analyzed. Gonadal fat mass in different genotypes of 12 weeks-old Ocn_{BW} mice (**D**). $n = 7$ mice per genotype analyzed. (**E–G**) Serum Osteocalcin levels in different genotypes of 12 weeks-old (**E**) and 5 weeks-old (**F**) Ocn_{GK} mice. $n = 11$ or more mice per genotype analyzed. Osteocalcin levels in different genotypes of 12 weeks-old Ocn_{BW} mice (**G**). $n = 4$ or more mice per genotype analyzed. (**H, I**) Fed (**H**) and fasting (**I**) glucose levels in Ocn_{BW} WT and WT129 mice. $n = 14$ or more mice per genotype analyzed. In bar plots, each dot represents an individual mouse. All data are shown as mean ± SEM. Statistical significance was determined by one-way Kruskal–Wallis test followed by post hoc multiple comparisons test. $*p < 0.05$; $**p < 0.01$, $****p < 0.0001$.
Source data are available online for this figure.

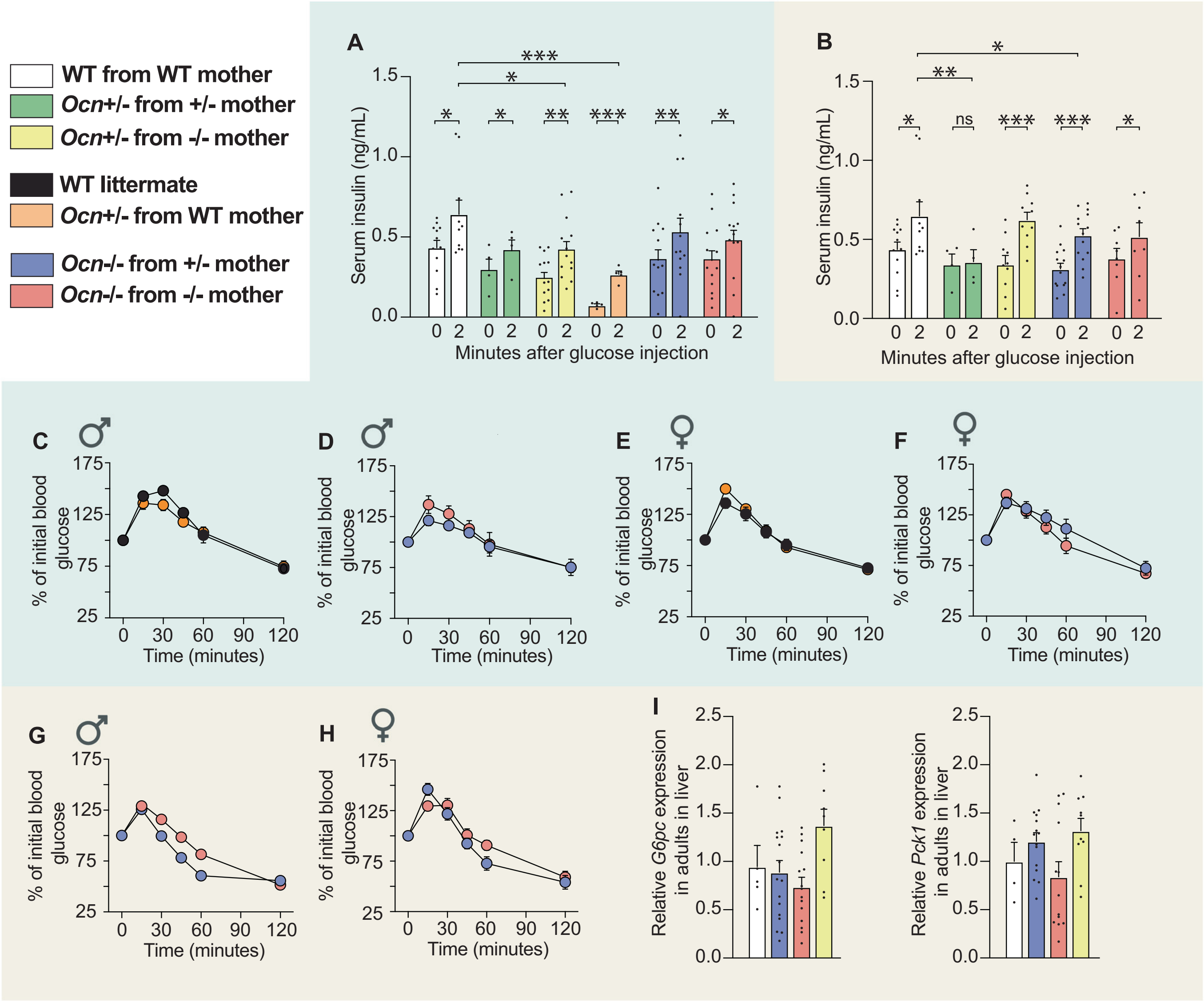

Figure EV2. Dynamic analysis of glucose metabolism in *Ocn-deficient* mouse strains.

(**A**, **B**) Serum insulin levels at 0 and 2 min after glucose injection in glucose stimulated insulin secretion test in mice of indicated genotypes of Ocn_{GK} mouse strain (**A**). $n = 4$ or more mice per genotype analyzed. Serum insulin levels at 0 and 2 min after glucose injection in glucose stimulated insulin secretion test in mice of indicated genotypes of Ocn_{BW} (**B**) mouse strain. $n = 7$ or more mice per genotype analyzed. (**C–H**) Serum glucose levels at different time points post injection of pyruvate in a pyruvate tolerance test in mice of indicated genotypes in the Ocn_{GK} (**C–F**) mouse strain. Serum glucose levels at different time points post injection of pyruvate in a pyruvate tolerance test in mice of indicated genotypes in Ocn_{BW} (**G**, **H**) mouse strain. $n = 6$ or more mice per genotype analyzed. (**I**) Relative Pck1 and G6pc expression in the liver from adult mice of different genotypes in the Ocn_{BW} strain. $n = 4$ or more mice per genotype analyzed. In bar plots, each dot represents an individual mouse. All data are shown as mean ± SEM. Statistical significance was determined by one-way Kruskal–Wallis test followed by post hoc multiple comparisons test. *$p < 0.05$; **$p < 0.01$; ***$p < 0.001$ ns: not significant. Source data are available online for this figure.

 © The Author(s)

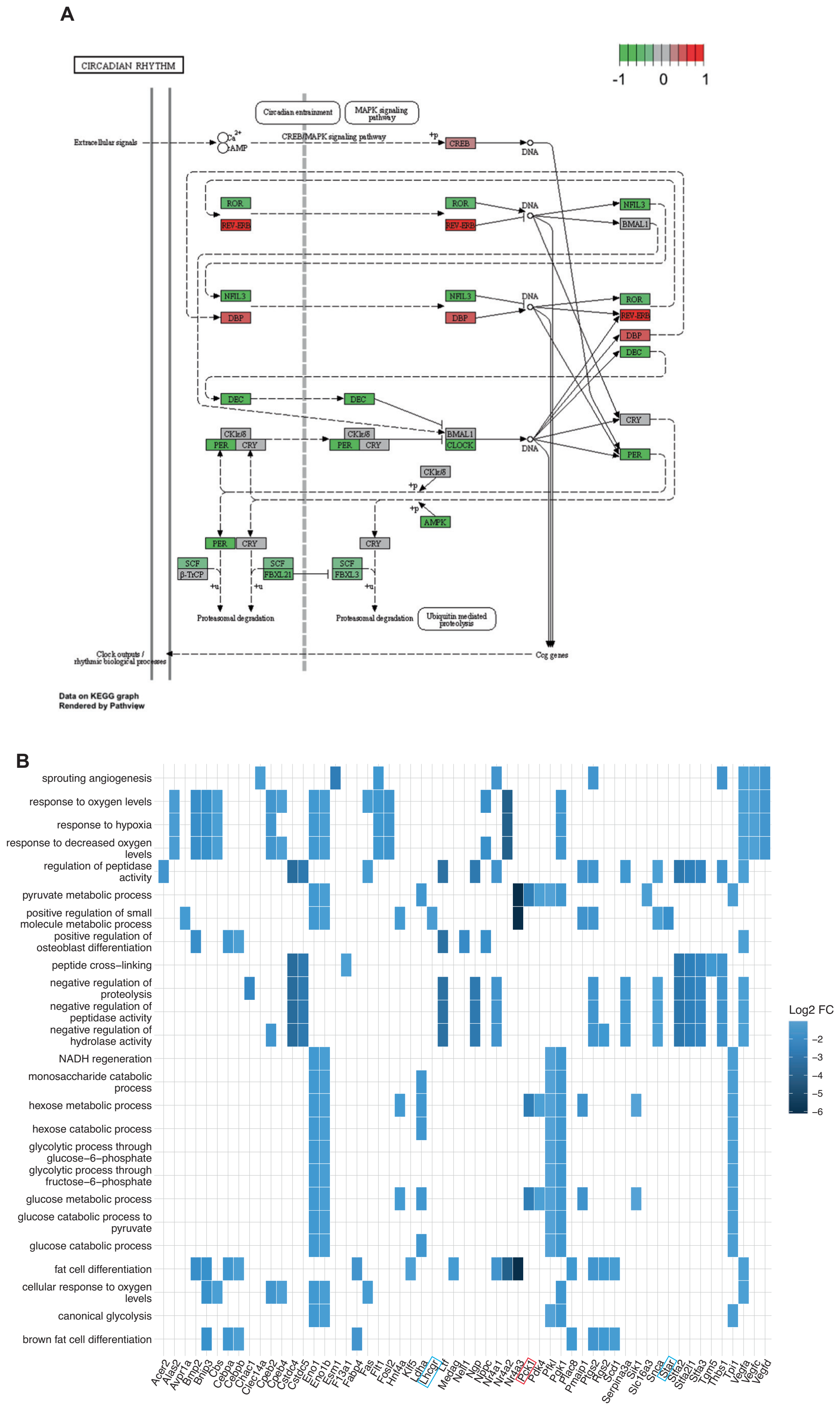
A
CIRCADIAN RHYTHM
-1 0 1
Circadian entrainment
MAPK signaling pathway
Extracellular signals
Ca2+
cAMP
CREB/MAPK signaling pathway
CREB
DNA
ROR
REV-ERB
NFIL3
BMAL1
DBP
DEC
CRY
PER
CKIε/δ
CLOCK
AMPK
SCF
β-TrCP
FBXL21
FBXL3
Proteasomal degradation
Ubiquitin mediated proteolysis
Clock outputs / rhythmic biological processes
Ccg genes
Data on KEGG graph
Rendered by Pathview
B
sprouting angiogenesis
response to oxygen levels
response to hypoxia
response to decreased oxygen levels
regulation of peptidase activity
pyruvate metabolic process
positive regulation of small molecule metabolic process
positive regulation of osteoblast differentiation
peptide cross−linking
negative regulation of proteolysis
negative regulation of peptidase activity
negative regulation of hydrolase activity
NADH regeneration
monosaccharide catabolic process
hexose metabolic process
hexose catabolic process
glycolytic process through glucose−6−phosphate
glycolytic process through fructose−6−phosphate
glucose metabolic process
glucose catabolic process to pyruvate
glucose catabolic process
fat cell differentiation
cellular response to oxygen levels
canonical glycolysis
brown fat cell differentiation
Log2 FC
−2
−3
−4
−5
−6
Acer2
Alas2
Avpr1a
Bmp2
Bnip3
Cbs
Cebpa
Cebpb
Chac1
Clec14a
Cpeb2
Cpeb4
Cstdc4
Cstdc5
Eno1
Eno1b
Esm1
F13a1
Fabp4
Fas
Flt1
Fosl2
Hnf4a
Klf5
Ldha
Lhcgr
Lif
Medag
Nell1
Ngp
Nppc
Nr4a1
Nr4a2
Nr4a3
Pck1
Pdk4
Pfkl
Pgk1
Plac8
Pmaip1
Ptgs2
Rgs2
Scd1
Serpina3a
Sik1
Slc16a3
Snca
Star
Stfa2
Stfa2l1
Stfa3
Tgm5
Thbs1
Tpi1
Vegfa
Vegfc
Vegfd

Figure EV3. KEGG analysis of transcriptomic studies of testes gene expression.

(**A**) KEGG pathview graph of the molecular clock comparing the transcriptomic data obtained from *Ocn*−/− mice to those from WT mice. Values close to −1 (green) indicate a downregulation of the gene in *Ocn*−/− compared to WT, values close to 0 (gray) indicate no difference and values close to 1 (red) indicate an upregulation in *Ocn*−/− compared to WT. (**B**) Heatplots of the top 25 GO biological processes (GO term) that are significantly downregulated in both *Ocn*+/− and *Ocn*−/− compared to WT mice. $n = 2$-3 mice per genotype analyzed.

© The Author(s)

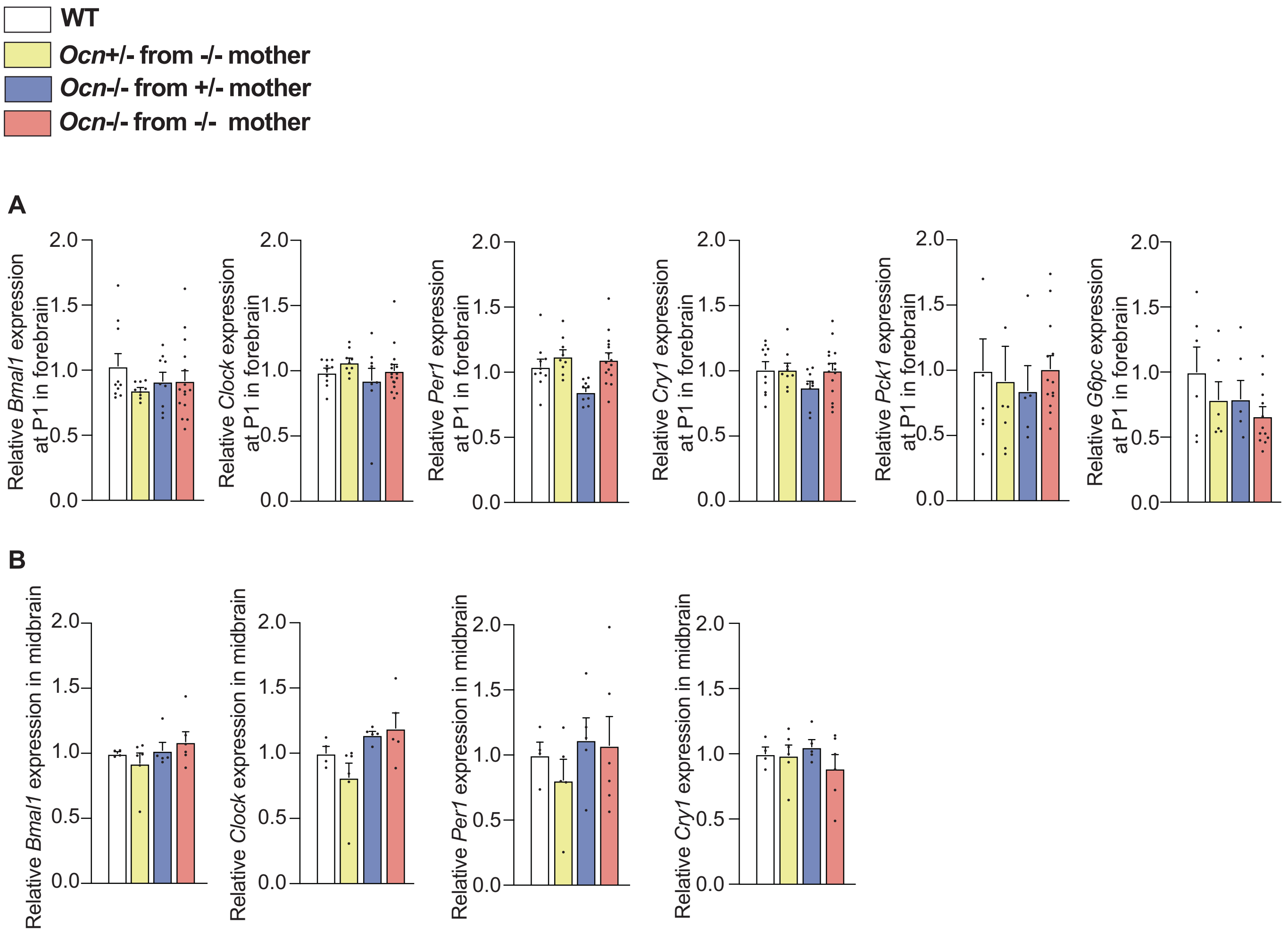

Figure EV4. Gene expression analysis in forebrain (newborn) and midbrain (adults).

(**A**) Relative expressions of circadian rhythm-related genes, Bmal1, Clock, Per1, and Cry1, and glucose homeostasis genes, Pck1 and G6pc in forebrain at P1 in different genotypes from Ocn_{GK} mouse strain. $n = 9$ or more mice per genotype analyzed. (**B**) Relative expressions of circadian rhythm-related genes Bmal1, Clock, Per1 and Cry1 in midbrain of adult mice of different genotypes from Ocn_{GK} mouse strain. $n = 4$ or more mice per genotype analyzed. In bar plots, each dot represents an individual mouse. All data are shown as mean ± SEM. Statistical significance was determined by one-way Kruskal–Wallis test followed by post hoc multiple comparisons test. Source data are available online for this figure.

© The Author(s)
